# Triazole-Based Thiamine Analogues as Inhibitors of Thiamine Pyrophosphate-Dependent Enzymes: 1,3-Dicarboxylate for Metal Binding

**DOI:** 10.1021/acsomega.4c04594

**Published:** 2024-09-30

**Authors:** Terence C. S. Ho, Alex H. Y. Chan, Finian J. Leeper

**Affiliations:** Yusuf Hamied Department of Chemistry, University of Cambridge, Lensfield Road, Cambridge CB2 1EW, U.K.

## Abstract

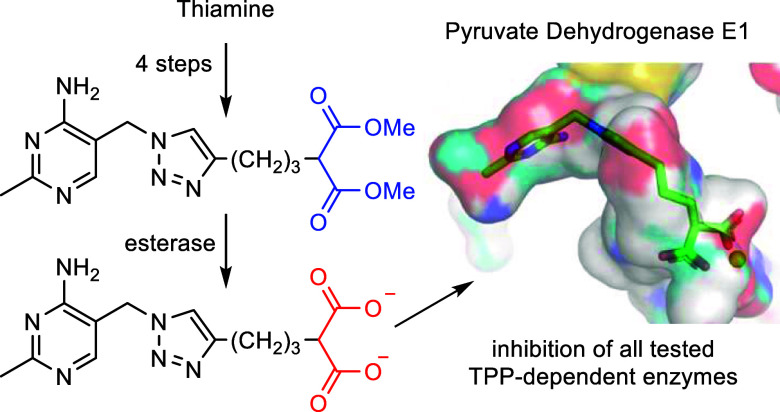

Thiamine **1** (vitamin B1) is essential for energy metabolism, and interruption of its utilization pathways is linked to various disease states. Thiamine pyrophosphate **2a** (TPP, the bioactive form of **1**) functions as a coenzyme of a variety of enzymes. To understand the role of vitamin B1 in these diseases, a chemical approach is to use coenzyme analogues to compete with TPP for the enzyme active site, which abolishes the coenzyme function. Exemplified by oxythiamine **3a** and triazole hydroxamate **4**, chemical probes require the coenzyme analogues to be membrane-permeable and of broad inhibitory activity to the enzyme family (rather than being too selective to particular TPP-dependent enzymes). In this study, using biochemical assays, we show that changing the hydroxamate metal-binding group of **4** to a 1,3-dicarboxylate moiety leads to the potent inhibition of multiple TPP-dependent enzymes. We further demonstrate that this dianionic thiamine analogue when masked in its diester form becomes membrane-permeable and can be unmasked by esterase treatment. Taken together, our inhibitors are potentially useful chemical tools to study the roles of vitamin B1, using a prodrug mechanism, to induce the effects of thiamine deficiency in cell-based assays.

## Introduction

**Figure 1:**
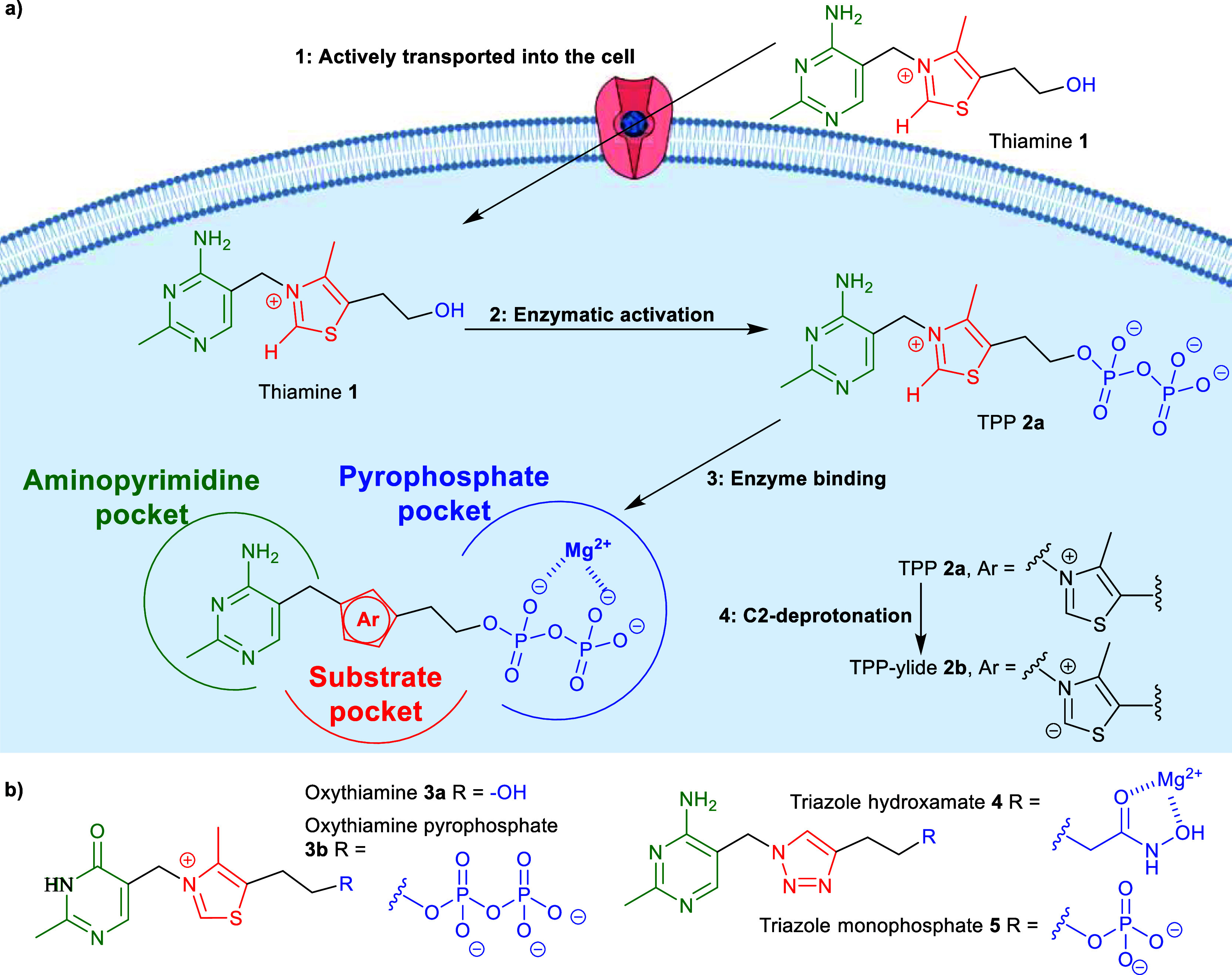
(a) Thiamine-utilization pathway in mammalian cells. (b) Structures of some coenzyme analogues. Created using “Icon Pack–Membrane” template with permission from BioRender.com (2024), https://app.biorender.com/biorender-templates.

Thiamine **1** (vitamin B1) is essential for energy metabolism, and its deficiency leads to neurological disorders.^1−3^ Being positively charged, **1** requires transport into the cytoplasm, where it is converted into the coenzyme thiamine pyrophosphate (TPP) **2a** by thiamine pyrophosphokinase (TPK) (Figure 1a).^1−4^ This polyanionic tail anchors TPP to the active sites of TPP-dependent enzymes by forming strong ionic interactions with a Mg^2+^ cation in the pyrophosphate pocket. The C2-position of the thiazolium ring is deprotonated (facilitated by the aminopyrimidine moiety) to yield TPP-ylide **2b**, which is the catalytically active species across the whole TPP-dependent enzyme family,^4^ which includes pyruvate dehydrogenase E1-subunit (PDH E1), pyruvate decarboxylase (PDC), pyruvate oxidase (PO), and oxoglutarate dehydrogenase E1-subunit (OGDH E1). Individual enzymes vary in substrate preferences and reactions catalyzed, but all share similar TPP-binding sites.^4^ Interruption of the thiamine-utilization pathway can result in neurodegeneration and is also linked with diabetes and found in many cancers.^1−3^ To help in understanding these diseases, compounds causing thiamine deficiency within the cells can be used.

A common method to study the coenzyme role of thiamine is to use thiamine/TPP analogues as TPP-competitive inhibitors of TPP-dependent enzymes.^5−18^ Among the reported analogues, most inhibitors are either potent but membrane-impermeable^5,6^ (thus not applicable in cell-based studies) or membrane-permeable but too selective toward particular TPP-dependent enzymes^7−14^ (more suitable for pharmaceutical or agricultural use). To study the global effects of vitamin B1 in cells, chemical tools used to induce thiamine deficiency should be able to suppress multiple TPP-dependent enzymes. There are two types of analogue that fulfill this requirement (Figure 1b).^15−17^ The first type, represented by oxythiamine **3a**, is a thiamine analogue bearing a modified pyrimidine ring. **3a** is a prodrug; it enters cells via thiamine transporters and has its metal-binding group (MBG) installed by TPK; the resultant oxythiamine pyrophosphate **3b** competes with TPP for the coenzyme-binding pocket of enzymes. C2-deprotonation is prevented by its “unnatural” pyrimidine ring and so the respective ylide is not formed. Occupying the coenzyme pocket but being catalytically inactive, **3b** is a TPP-competitive inhibitor of the enzyme family.^15,16^ The second type of analogue, represented by triazole hydroxamate **4**, possesses a neutral MBG in place of the pyrophosphate and an unnatural central ring, with the positive thiazolium ring of TPP replaced by a neutral triazole; the latter abolishes the catalytic activity. By capturing the strong stabilizing interactions between the enzyme’s hydrophobic region and the TPP-ylide **2b**, this neutral scaffold (at least partially) compensates for the weakened interactions with the Mg^2+^ cation.^17^ While **3a** is widely used to induce thiamine deficiency in cells and in vivo,^1,15^ we have recently established **4** as a competent chemical probe for the vitamin B1 pathway in cell-based studies.^17^

In this study, we develop a new series of TPP-competitive inhibitors showing broad inhibitory activities to the enzyme family. This novel type of analogue is structurally similar to **4** (both featuring the aminopyrimidine-CH_2_–triazole motif) but mechanistically similar to **3** (both involving an enzymatic activation step to install an anionic MBG on the tail). We envision that the coenzyme analogues from this work have the potential to be useful tools to study the roles of vitamin B1, complementing **3a** and **4** in inducing the effects of thiamine deficiency in cell-based assays.

## Results and Discussion

The ligand design in this study came from the recent discovery^5^ of triazole monophosphate **5** (Figure 1b) as an inhibitor of multiple TPP-dependent enzymes. However, **5** is unlikely to be a useful cellular probe due to its anionic phosphate moiety.^6,18−20^ Since membrane permeability is often a limiting factor in the application of coenzyme analogues in cell-based studies, compounds developed in this study had their polarity/permeability estimated based on the calculated Log *D* at pH 7.4 (*c* Log *D*) and the parallel artificial membrane permeability assay (PAMPA)^21^ (refer to Table S2 for details). In line with its *c* Log *D* of −3.8, **5** was found to be membrane-impermeable in PAMPA (fraction absorbed = 2%). Despite this, we envisioned that the broad inhibitory action against the TPP-dependent enzyme family rendered **5** as a good starting point for probe development.

**Scheme 1:**
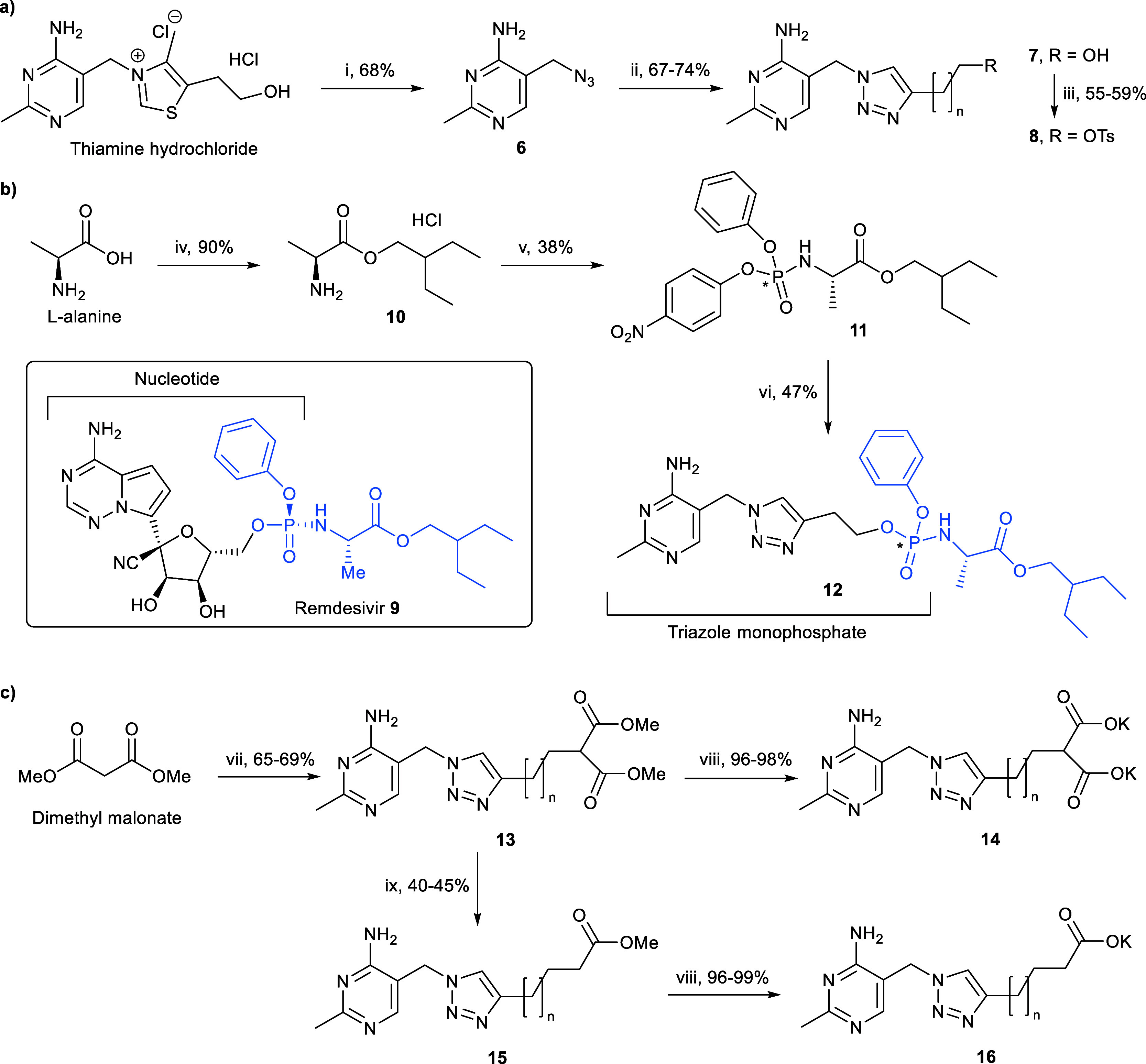
(a) Synthesis of Precursors **7** and **8**.^17^ (b) Synthesis of **12**; * Indicates a Diastereomeric Mixture (at the Phosphorus Centre) in **11** and **12**. (c) Synthesis of **13**–**16** Reagents and conditions: (i) NaN_3_, Na_2_SO_3_, water, 65 °C; (ii) 3-butyn-1-ol or 4-pentyn-1-ol, CuSO_4_ 5H_2_O, sodium ascorbate, *t*-BuOH, water, RT; (iii) *p*-TsCl, pyridine, RT; (iv) thionyl chloride, 2-ethylbutanol, 60 °C; (v) OP(OPh)Cl_2_, DIPEA, DCM, −78 °C, then 4-nitrophenol, DIPEA, 0 °C; (vi) **7a**, MgCl_2_, DIPEA, MeCN, 50 °C; (vii) **8**, NaH, DMF, 40 °C; (viii) 1 M aq. KOH, THF, water, 30 °C; (ix) LiCl, wet DMSO, 125 °C. For all compounds: **a***n* = 1, **b***n* = 2.

In this study, the triazole scaffold was retained due to its ease of synthesis (Scheme 1a).^17^ In contrast to the diphosph(on)ate moiety, which has limited options for prodrug development,^19^ various systems that mask monophosph(on)ate groups have been developed, and many phosphate prodrugs have gained approval for clinical applications.^18^ Inspired by the life-saving COVID-19 antiviral agent remdesivir **9**,^20^ we incorporated its progroup onto our triazole scaffold (**7a**). As shown in Scheme 1b, coupling between **11** (obtained in two steps from l-alanine)^20^ and **7a** yielded prodrug **12**, a protected version of **5**. **12** is reasonably hydrophobic with a cLogD value of 3.9 and was found to be membrane-permeable in PAMPA (fraction absorbed = 48%). Under the assay conditions given in our previous study, the inhibitory concentration (IC_50_) values of **5** for PDH E1, PDC, PO, and OGDH E1 were 6.6, 1300, 348, and 170 μM, respectively.^5^ Under the same assay conditions with **12** at 10 and 200 μM, respectively, no inhibition was observed on PDH and OGDH, while **12** was not sufficiently soluble under the assay conditions used for PDC and PO (1300 and 400 μM). The lack of inhibition on PDH and OGDH was expected and attributed to both the electronic and steric factors: the neutral phosphate progroup cannot form ionic interactions with the Mg^2+^ cation and is too bulky to be accommodated in the pyrophosphate pocket. Further testing of **12** at 100 μM on PDH E1 still resulted in no inhibition, and it was insufficiently soluble for testing at 300 μM on OGDH E1.

Collectively, the above data on **12** suggested that the phosphate progroup in remdesivir **9** could be adapted to our triazole scaffold, masking the metal-binding capability of **5**, and rendering it membrane-permeable. Despite the promising data, the poor solubility in aqueous environments (<300 μM in PBS with 5% DMSO) would limit its use in cell-based studies, and so we did not conduct further biological characterization of **12**.

The development of prodrug **12** from inhibitor **5**, though it did not lead to a useful chemical tool, established that dianionic thiamine analogues can still be made membrane-permeable as long as a hydrophobic progroup is appropriately chosen to mask the charged MBG. Many of the prodrugs currently in clinical use are esters and are activated by esterases:^22^ remdesivir is itself an example of this. This prompted us to explore 1,3-dicarboxylate as the MBG where the two carboxylate groups can chelate the metal ion in a six-membered ring. The corresponding diesters could then be the prodrugs. Dicarboxylates **14a** and **b** were chosen because docking models suggested that linkers shorter than **14a**′s or longer than **14b**′s would suboptimally position the MBG in the pyrophosphate pocket. The chosen progroup is a methyl ester. As shown in Scheme 1c, diesters **13a** and **b** could be prepared from tosylates **8a** and **b**, respectively, by reaction with dimethyl malonate and NaH. Subsequent basic hydrolysis yielded dicarboxylates **14a** and **b**. To show that the strength of interactions between the MBG and the Mg^2+^ cation increases with the negative charges on the tail, monocarboxylates **16a** and **b** were also prepared. Krapcho decarbomethoxylation^23^ of diesters **13a** and **b** gave monoesters **15a** and **b**, which were hydrolyzed under basic conditions to yield monocarboxylates **16a** and **b**.

**Figure 2:**
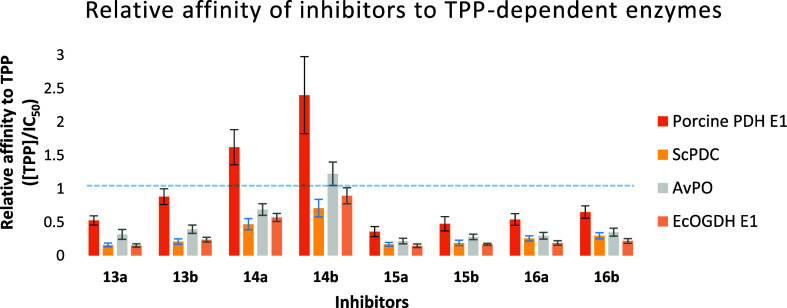
Histograms showing the relative affinity (relative to TPP, i.e., ^[TPP]^/_IC_50__) of each inhibitor for each of the four different enzymes; compounds above the dotted line have IC_50_ values lower than the TPP concentration used, which means they have greater affinity than TPP.

To demonstrate broad-spectrum inhibition of TPP-dependent enzymes, four enzymes across three kingdoms were selected, namely, porcine PDH E1, *Saccharomyces cerevisiae* PDC, *Aerococcus viridans* PO, and *Escherichia coli* OGDH E1. The different biochemical reactions catalyzed, preferred substrates (Table S1), and primary sequences reflect the structurally and functionally diverse members of the family. As summarized in Table S1, preliminary screening with compounds **13–16a,b** at a concentration 5-fold greater than that of TPP showed that our analogues are inhibitors of all the TPP-dependent enzymes (percentage inhibition >38%). Screening at increased TPP levels where [compound] = [TPP] resulted in reduced apparent inhibition in all cases, confirming that the analogues are competitive with the TPP coenzyme. Further biochemical study showed that our analogues inhibited the enzymes in a dose-dependent manner (Figures S1–S4), and their IC_50_ values are summarized in Table S1. However, as the inhibitors are TPP-competitive, what really matters is the affinity of each inhibitor relative to the affinity of TPP for that particular enzyme. Figure 2, therefore, presents this “relative affinity” (=^[TPP]^/_IC_50__) for each of the eight compounds against each of the four enzymes. This metric also shows which of the four enzymes are inhibited more and which are inhibited less at any particular concentration of inhibitor and of TPP.

**Figure 3:**
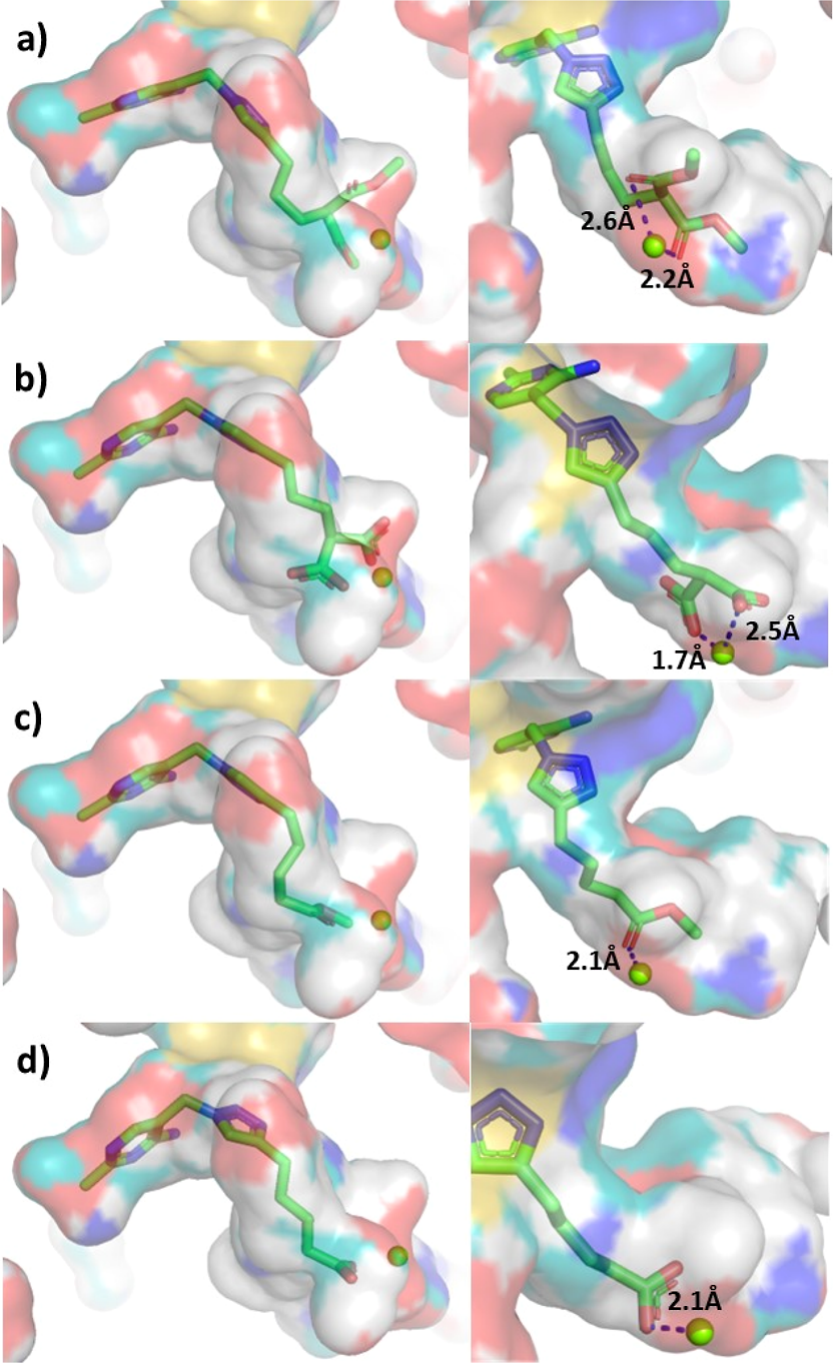
Predicted binding modes of (a) **13b**, (b) **14b**, (c) **15b**, and (d) **16b** (green carbon atoms) in the active site (shown as surface) of human PDH E1 (PDB: 6CFO). Left column: the binding modes of the inhibitors are similar to that of TPP, with a V-shaped conformation between aminopyrimidine and the thiazolium ring. Right column: views showing the interactions between the MBG and the Mg^2+^ cation. The Mg^2+^ is represented as a yellowish-green sphere.

Comparison of the relative affinities (Figure 2) shows that affinities increase with the number of negative charges of the tail due to the electrostatic interactions with the Mg^2+^ cation in the pyrophosphate pocket, consistent with our previous findings.^5^ The longer homologues bind more tightly than their shorter counterparts regardless of the type of tail, with the dicarboxylates showing the greatest difference. The higher sensitivity to chain-length in the dicarboxylates might be attributed to their bidentate metal-binding geometry, in contrast to the monodentate MBG in monoesters and monocarboxylates (Figures 3 and S5). Diesters **13** could be regarded as a protected version of dicarboxylates **14**, and computational dockings predicted that **13a** and **b** rely on the carbonyl groups of their two esters to form polar interactions with the Mg^2+^ cation (Figure 3). Collectively, dicarboxylate **14b** is the most potent TPP-competitive inhibitor, with its affinities comparable to those of the natural coenzyme TPP; diester **13b** and monocarboxylate **16b** are similarly potent, with affinities approximately 3-fold lower than those of **14b**; and monoester **15b** is the weakest inhibitor in this series.

Dicarboxylate **14b** was found to be membrane-impermeable in PAMPA (fraction absorbed = 1%), consistent with its *c* Log *D* of −4.5. By contrast, its protected version (**13b**) was hydrophobic enough (*c* Log *D* = 0.8) to permeate biological membranes (fraction absorbed = 42%, PAMPA data summarized in Table S2). Other esters (**13a**, **15a**, and **15b**) were shown to be membrane-permeable, whereas other carboxylates (**14a**, **16a**, and **16b**) were impermeable, as expected.

**Figure 4:**
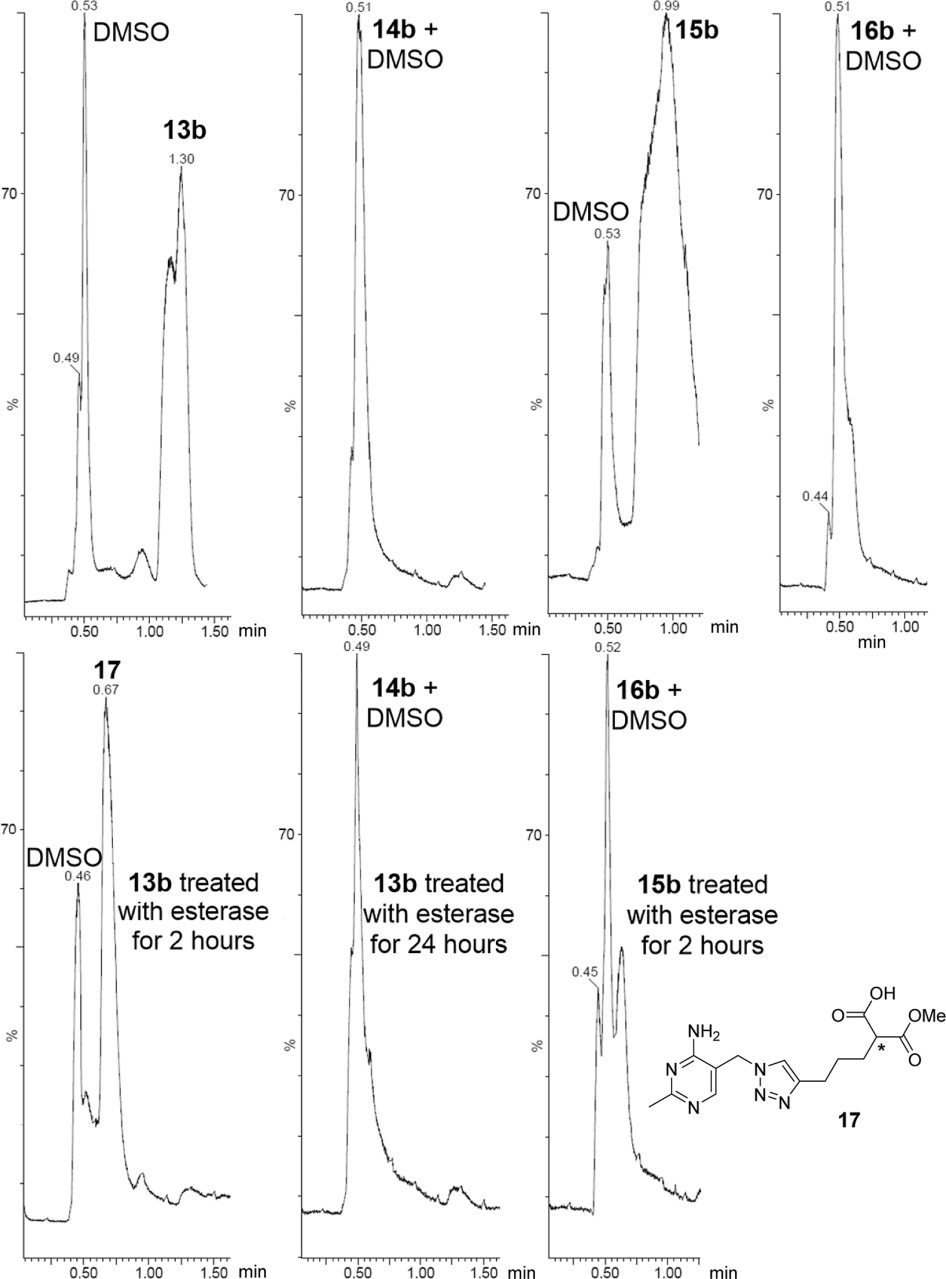
UPLC-HRMS analysis of the esterase treatment. The vertical axis shows total ion current as a percentage of the highest peak. Compound (retention time/min): diester **13b** (1.30), dicarboxylate **14b** (0.51), monoester **15b** (0.99), monocarboxylate **16b** (0.51), and intermediate **17** (0.67). Top row: UPLC analysis of the pure compounds (without esterase treatment); bottom row: UPLC analysis of the reaction mixture after esterase treatment for the duration indicated (these chromatographs also showed broad peaks in the region 2.5–6.5 min due to the enzyme).

Lastly, the feasibility of progroup cleavage to unveil the corresponding charged MBG was biochemically demonstrated. Esters **13b** and **15b** were subjected to esterase treatment (porcine liver esterase) at 37 °C and pH 7.4 for 2 h, and then the reaction mixtures were analyzed using UPLC-HRMS (Figure 4).^24^ The molecular mass of the new products formed was determined by MS and confirmed that they were the expected carboxylic acids. Monoester **15b** was completely hydrolyzed into monocarboxylate **16b**, but diester **13b** underwent a two-step hydrolysis; after 2 h; the major product was monoester monocarboxylate **17,** but after 24 h, the only product seen was dicarboxylate **14b**. The second hydrolysis is presumably slower because the substrate **17** is anionic, whereas for the first hydrolysis, the substrate **13b** is neutral.^19,25,26^ When **13b** enters a cell through passive diffusion, the intracellular esterases will begin to hydrolyze the ester groups to give first **17** and then **14b**. Since both **17** and **14b** are negatively charged under physiological conditions, they will be unable to diffuse back out of the cell and so should accumulate there.^27^ Diester **13b** is more hydrophobic than triazole hydroxamate **4** (*c* Log *D*: 0.8 vs −0.5) and so should diffuse into cells more readily. After hydrolysis, **14b** has binding affinities (for TPP-dependent enzymes) comparable to or better than those of **4**. Thus, we anticipate that **13b** will be as useful as **4** in probing the role of vitamin B1 in cells.

## Conclusions

Coenzyme analogues, such as oxythiamine **3a** and triazole hydroxamate **4** which are TPP-competitive enzyme inhibitors, have been used to induce thiamine deficiency in cell-based assays and to study the roles of vitamin B1 in diseases. To be qualified as chemical probes for the vitamin B1 pathway, compounds must be membrane-permeable and show broad inhibitory activities against TPP-dependent enzymes. As all TPP-dependent enzymes hold a divalent metal ion in the pyrophosphate pocket to bind the coenzyme, ligands aimed to target multiple TPP-dependent enzymes are often designed to possess an MBG on the tail. Coenzyme analogues featuring an anionic MBG are usually more potent than those with a neutral MBG; however, the negatively charged analogues are often poorly membrane-permeable. In this work, we adopted a prodrug strategy to bypass this issue. Our biochemical studies revealed that coenzyme analogues **5** and **14b** both suppress multiple TPP-dependent enzymes, and our computational study predicted that they used monophosphate and 1,3-dicarboxylate, respectively, as the MBG. Their corresponding protected versions (**12** and **13b**) have been chemically synthesized and were shown to be membrane-permeable. Subjected to esterase treatment, diester **13b** was successfully hydrolyzed to dicarboxylate **14b**, which is a more potent TPP-competitive inhibitor. Diester **13b** also has the advantage over oxythiamine **3a** that it does not have to compete with thiamine for its transport into cells and its subsequent activation, which is a limitation for **3a**. However, a limitation of our probe is that while hydrolysis of **13b** to **17** occurs readily, complete hydrolysis to **14b** is slow. We envision that altering the structure of our diester, to optimize the kinetics of prodrug activation, will further enhance the applicability of our tool.

The affinities of dicarboxylate **14b** for the various TPP-dependent enzymes are all similar to the affinities of TPP itself, ranging from 2.4 times the affinity for PDH E1 down to 0.7 times for PDC. This is better than hydroxamate **4**, which ranges from 1.49 down to 0.25,^17^ and better still than monophosphate **5**,^5^ which ranges from 1.5 down to 0.11. Comparing the percentage inhibition for **14b** with the values previously obtained^17^ for oxythiamine pyrophosphate **3b**, **14b** is more potent against PO and OGDH and similarly potent against PDH. The affinities could be further improved by changing from the triazole central ring to a more hydrophobic ring, e.g., thiophene, which, in general, gives ca. 3–5 times tighter binding.^5^ However, the synthesis of such a compound would be much more complicated.

All together, we can conclude that easily synthesized diester **13b** has the potential to be a useful chemical tool to induce thiamine deficiency by displacing the coenzyme from the active sites of TPP-dependent enzymes in general, after hydrolysis to **14b**.

## Experimental Methods

### Enzyme Inhibitory Activity Assays

#### Porcine PDH E1 Inhibitory Activity Assay

Porcine PDH E1 was purchased from Sigma. Porcine PDH E1 activity was determined by monitoring 2,6-dichlorophenolindophenol (DCPIP) reduction at 600 nm using a microplate reader (CLARIOstar) and conducted as described^5^ with some modifications. The percentage inhibition of compounds against porcine PDH E1 was assayed at 250 μM. The reaction buffer (50 mM KH_2_PO_4_ and 1 mM MgCl_2_, pH 7) contained thiamine pyrophosphate (TPP) at specified concentrations, 0.25 mM DCPIP, and 2 mg/mL porcine PDH E1. The reaction mixture was preincubated at 37 °C with the inhibitor for 30 min, and then the reaction was initiated by adding pyruvate to a final concentration of 50 mM. To determine the half-maximal inhibitory concentration (IC_50_), the TPP concentration was set at 60 μM with varying inhibitor concentrations. Specific activity was calculated using the molar extinction coefficient of DCPIP, 21 mM^–1^ cm^–1^.^28^ The IC_50_ values were calculated from nonlinear regression curve fitting using GraphPad Prism. The compound affinity (*K*_I_) values were obtained by comparison to the affinity of TPP; *K*_M(TPP)_ was found to be 0.05 μM, consistent with the value previously reported.^7^

#### *S. cerevisiae* PDC Inhibitory Activity Assay

*S. cerevisiae* PDC was purchased from Sigma. *S. cerevisiae* PDC activity was determined by monitoring DCPIP reduction at 600 nm using a microplate reader (CLARIOstar) and conducted as described^5^ with some modifications. The percentage inhibition of compounds against *S. cerevisiae* PDC was assayed at 750 μM. The reaction buffer (50 mM KH_2_PO_4_ and 1 mM MgCl_2_, pH 7) contained TPP at specified concentrations, 0.27 mM DCPIP, and 0.15 mg/mL *S. cerevisiae* PDC. The reaction mixture was preincubated at 37 °C with the inhibitor for 60 min, and then the reaction was initiated by adding pyruvate to a final concentration of 70 mM. To determine the IC_50_, TPP concentration was set at 150 μM with varying inhibitor concentrations. Specific activity was calculated using the molar extinction coefficient of DCPIP, 21 mM^–1^ cm^–1^.^28^ The IC_50_ values were calculated from nonlinear regression curve fitting using GraphPad Prism. The *K*_I_ values were obtained by comparison to *K*_M(TPP)_, which was found to be 15 μM, consistent with the value previously reported.^7^

#### *A. viridans* PO Inhibitory Activity Assay

*A. viridans* PO and horseradish peroxidase were purchased from Sigma. *A. viridans* PO activity was determined by monitoring appearance of quinoneimine dye at 550 nm using a microplate reader (CLARIOstar) and conducted as described^5^ with some modifications. The percentage inhibition of compounds against *A. viridans* PO was assayed at 250 μM. The reaction buffer (50 mM KH_2_PO_4_ and 10 mM MgCl_2_, pH 5.9) contained TPP at specified concentrations, 10 μM flavin adenine dinucleotide, 0.15% 4-aminoantipyrine, 0.3% *N*-ethyl-*N*-(2-hydroxy-3-sulfopropyl)-m-toluidine (EHSPT), 50 μg/mL horseradish peroxidase, and 0.35 U/mL *A. viridans* PO. The reaction mixture was preincubated at 37 °C with inhibitor for 30 min, and then the reaction was initiated by adding pyruvate to a final concentration of 50 mM. To determine the IC_50_, TPP concentration was set at 60 μM with varying inhibitor concentrations. One unit of PO activity is defined as the production of 1 μmol of hydrogen peroxide per minute. The IC_50_ values were calculated from nonlinear regression curve fitting using GraphPad Prism. The *K*_I_ values were obtained by comparison to *K*_M(TPP)_, which was found to be 5 μM, consistent with the value previously reported.^5^

#### *E. coli* OGDH E1 Inhibitory Activity Assay

*E. coli* OGDH E1 was from material donated by R. Frank. *E. coli* OGDH E1 activity was determined by monitoring DCPIP reduction at 600 nm using a microplate reader (CLARIOstar) and conducted as described^5^ with some modifications. The percentage inhibition of compounds against *E. coli* OGDH E1 was assayed at 250 μM. The reaction buffer (50 mM KH_2_PO_4_ and 2 mM MgCl_2_, pH 7) contained TPP at specified concentrations, 0.5 mM DCPIP, and 6.7 mg/mL *E. coli* OGDH E1. The reaction mixture was preincubated at 37 °C with the inhibitor for 60 min, and then the reaction was initiated by adding α-ketoglutarate to a final concentration of 10 mM. To determine the IC_50_, the TPP concentration was set at 60 μM with varying inhibitor concentrations. Specific activity was calculated using the molar extinction coefficient of DCPIP, 21 mM^–1^ cm^–1^.^28^ The IC_50_ values were calculated from nonlinear regression curve fitting using GraphPad Prism. The *K*_*I*_ values were obtained by comparison to *K*_M(TPP)_, which was found to be 3 μM, consistent with the value previously reported.^7^

### General Procedure for Preparation of Diesters **13a** and **13b**

To a stirred solution of dimethyl malonate (0.24 mL, 2.0 mmol) in dry DMF (1.0 mL, 0.2 M) under nitrogen at 0 °C was added NaH (60% in mineral oil, 80 mg, 2.0 mmol). The resultant mixture was stirred at rt for 1 h, treated with tosylate **8**(17) (1.0 mmol), stirred at 40 °C for 3 days, quenched with aqueous phosphate buffer (pH 7) (30 mL), and extracted with *n*-BuOH (3 × 50 mL). The combined organic phases were dried over MgSO_4_, filtered, and evaporated under reduced pressure. The residue was purified by silica flash chromatography (5–15% MeOH in DCM) to yield diester **13**.

### General Procedure for Preparation of Monoesters **15a** and **15b**

To a stirred solution of diester **13** (0.6 mmol) in dry DMSO (3.0 mL, 0.2 M) at rt was added water (0.06 mL, 3.0 mmol) and LiCl (252 mg, 6.0 mmol). The resultant mixture was stirred at 125 °C overnight, diluted with *n*-BuOH (70 mL), washed with water (30 mL), dried over MgSO_4_, filtered, and evaporated under reduced pressure. The residue was purified by silica flash chromatography (5–15% MeOH in DCM) to yield monoester **15** as a solid.

### General Procedure for Preparation of Carboxylates **14a**, **14b**, **16a**, and **16b**

To a stirred solution of corresponding ester (0.2 mmol) in THF (0.5 mL) and water (0.5 mL) at rt was added 1 M aq. KOH (0.2 mL for **15** or 0.4 mL for **13**). The resultant mixture was stirred at 40 °C for 4 h and concentrated under reduced pressure to yield carboxylates **14** and **16** as solids.

## Supporting Information Available

Methods and results for enzyme assays, computational docking, and PAMPA; synthetic methods; compound characterization; and NMR spectra (PDF)
