# Supplementary material for: Triazole-Based Thiamine Analogues as Inhibitors of Thiamine Pyrophosphate-Dependent Enzymes: 1,3-Dicarboxylate for Metal Binding
Source: ACS Omega. 2024 Sep 30;9(41):42245–52. doi: 10.1021/acsomega.4c04594 (PMC11483378; doi:10.1021/acsomega.4c04594)
Supplement: Supplementary file 1 — ao4c04594_si_001.pdf [file ao4c04594_si_001.pdf]

Triazole-Based Thiamine Analogues as Inhibitors of Thiamine Pyrophosphate-Dependent Enzymes: 1,3-Dicarboxylate for Metal Binding

Terence C. S. Ho,[a‡] Alex H. Y. Chan[a‡] and Finian J. Leeper[a*]

[a] Yusuf Hamied Department of Chemistry, University of Cambridge, Lensfield Road, Cambridge CB2 1EW, UK.
[‡] Equal contribution.
*Corresponding author, e-mail address: fjl1@cam.ac.uk

Supplementary Information (SI)

**Enzyme inhibitory activity assays – methods and results (Figures S1-4)**	**S2**
**Summary of inhibitory activity on TPP-dependent enzymes (Table S1)**	**S7**
**Computational docking – methods and results (Figure S5)**	**S8**
**PAMPA – methods and results (Table S2)**	**S9**
**UPLC-HRMS analysis of the esterase treatment – methods**	**S10**
**Synthetic experimental procedures**	**S10**
**NMR spectra**	**S15**
**References**	**S33**

Enzyme inhibitory activity assays – methods and results (Figures S1-4)

Porcine PDH E1 inhibitory activity assay. Porcine PDH E1 was purchased from Sigma. Porcine PDH E1 activity was determined by monitoring 2,6-dichlorophenolindophenol (DCPIP) reduction at 600 nm using a microplate reader (CLARIOstar) and conducted as described[3] with some modifications. The percentage inhibition of compounds against porcine PDH E1 was assayed at 250 μM. The reaction buffer (50 mM KH_2PO_4 and 1 mM $MgCl_2$, pH 7) contained thiamine pyrophosphate (TPP) at specified concentrations, 0.25 mM 2,6-dichlorophenolindophenol (DCPIP), and 2 mg/mL porcine PDH E1. The reaction mixture was preincubated at 37 °C with the inhibitor for 30 min, then reaction was initiated by adding pyruvate to a final concentration of 50 mM. To determine the half-maximal inhibitory concentration (IC_{50}), TPP concentration was set at 60 μM with varying inhibitor concentrations. Specific activity was calculated using the molar extinction coefficient of DCPIP, 21 mM^{-1} cm^{-1}.[4] The IC_{50} values were calculated from non-linear regression curve fitting using GraphPad Prism. The compound affinity (K_I) values were obtained by comparison to the affinity of TPP (K_D); $K_{D(TPP)}$ was found to be 0.05 μM, consistent with the value previously reported.[3]

S. cerevisiae **PDC inhibitory activity assay.** *S. cerevisiae* PDC was purchased from Sigma. *S. cerevisiae* PDC activity was determined by monitoring DCPIP reduction at 600 nm using a microplate reader (CLARIOstar) and conducted as described[3] with some modifications. The percentage inhibition of compounds against *S. cerevisiae* PDC was assayed at 750 μM. The reaction buffer (50 mM KH_2PO_4 and 1 mM $MgCl_2$, pH 7) contained TPP at specified concentrations, 0.27 mM DCPIP, and 0.15 mg/ml *S. cerevisiae* PDC. The reaction mixture was preincubated at 37 °C with the inhibitor for 60 min, then reaction was initiated by adding pyruvate to a final concentration of 70 mM. To determine the IC_{50}, TPP concentration was set at 150 μM with varying inhibitor concentrations. Specific activity was calculated using the molar extinction coefficient of DCPIP, 21 mM^{-1} cm^{-1}.[4] The IC_{50} values were calculated from non-linear regression curve fitting using GraphPad Prism. The K_I values were obtained by comparison to $K_{D(TPP)}$ which was found to be 15 μM, consistent with the value previously reported.[3]

A. viridans **PO inhibitory activity assay.** *A. viridans* PO and horseradish peroxidase were purchased from Sigma. *A. viridans* PO activity was determined by monitoring appearance of quinoneimine dye at 550 nm using a microplate reader (CLARIOstar) and conducted as described[3] with some modifications. The percentage inhibition of compounds against *A. viridans* PO was assayed at 250 μM. The reaction buffer (50 mM KH_2PO_4 and 10 mM $MgCl_2$, pH 5.9) contained TPP at specified concentrations, 10 μM flavin adenine dinucleotide (FAD), 0.15% 4-aminoantipyrine, 0.3% N-ethyl-N-(2-hydroxy-3-sulfopropyl)-m-toluidine (EHSPT), 50 μg/mL horseradish peroxidase and 0.35 U/mL *A. viridans* PO. The reaction mixture was preincubated at 37 °C with inhibitor for 30 min, then reaction was initiated by adding pyruvate to a final concentration of 50 mM. To determine the IC_{50}, TPP concentration was set was set at 60 μM with varying inhibitor concentrations. One unit of PO activity is defined as the production of 1 μmol of hydrogen peroxide per minute. The IC_{50} values were calculated from non-linear regression curve fitting using GraphPad Prism. The K_I values were obtained by comparison to $K_{D(TPP)}$ which was found to be 5 μM, consistent with the value previously reported.[3]

E. coli **OGDH E1 inhibitory activity assay.** *E. coli* OGDH E1 was from material donated by R. Frank. *E. coli* OGDH E1 activity was determined by monitoring DCPIP reduction at 600 nm using a microplate reader (CLARIOstar) and conducted as described[3] with some modifications. The percentage inhibition of compounds against *E. coli* OGDH E1 was assayed at 250 μM. The reaction buffer (50 mM KH_2PO_4 and 2 mM $MgCl_2$, pH 7) contained TPP at specified concentrations, 0.5 mM DCPIP, and 6.7 mg/ml *E. coli* OGDH E1. The reaction mixture was preincubated at 37 °C with the inhibitor for 60 min, then reaction was initiated by adding α-ketoglutarate to a final concentration of 10 mM. To determine the IC_{50}, TPP concentration was set at 60 μM with varying inhibitor concentrations. Specific activity was calculated using the molar extinction coefficient of DCPIP, 21 mM^{-1} cm^{-1}.[4] The IC_{50} values were calculated from non-linear regression curve fitting using GraphPad Prism. The K_I values were obtained by comparison to $K_{D(TPP)}$ which was found to be 3 μM, consistent with the value previously reported.[3]

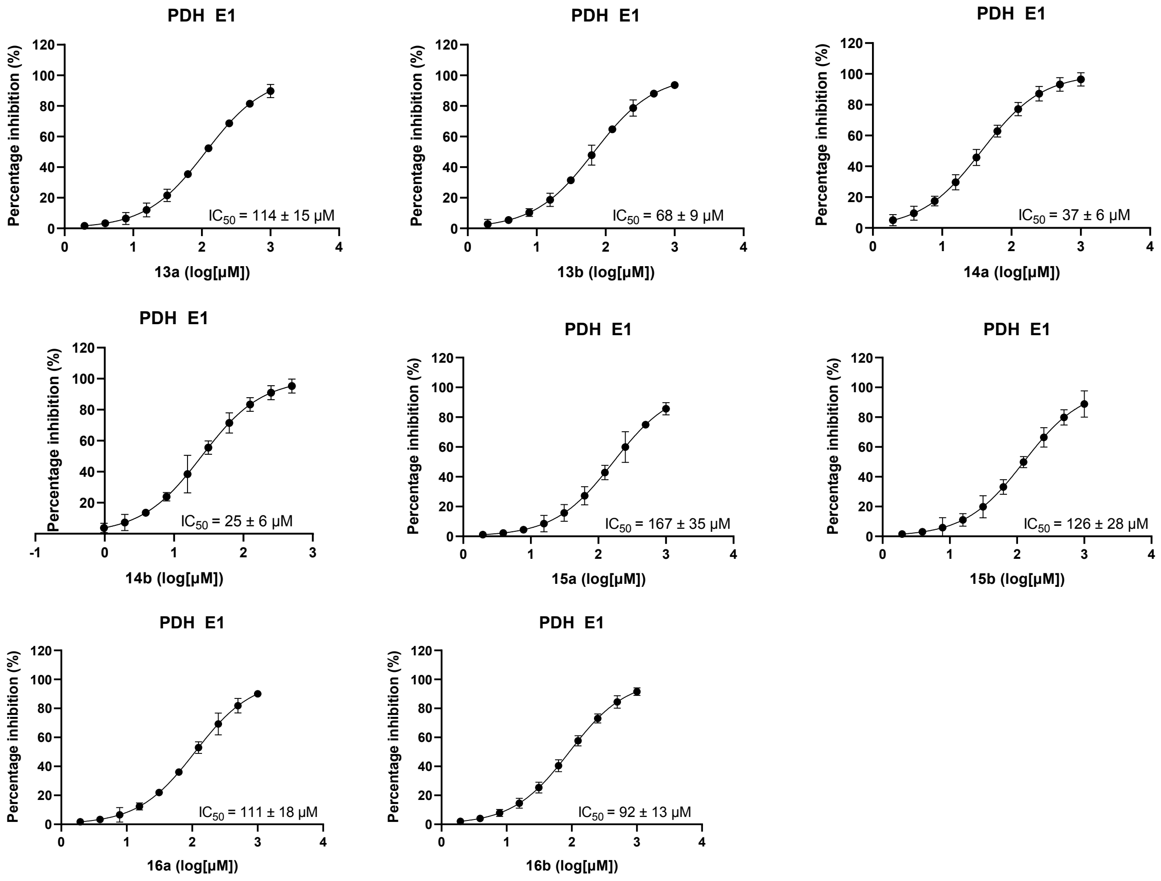

Figure S1. Measurement of porcine PDH E1 IC_{50} values at [TPP] = 60 µM. Measurements were made in triplicate. Where the error bars are not visible, they are smaller than the symbols. Best-fit nonlinear regression curves are shown.

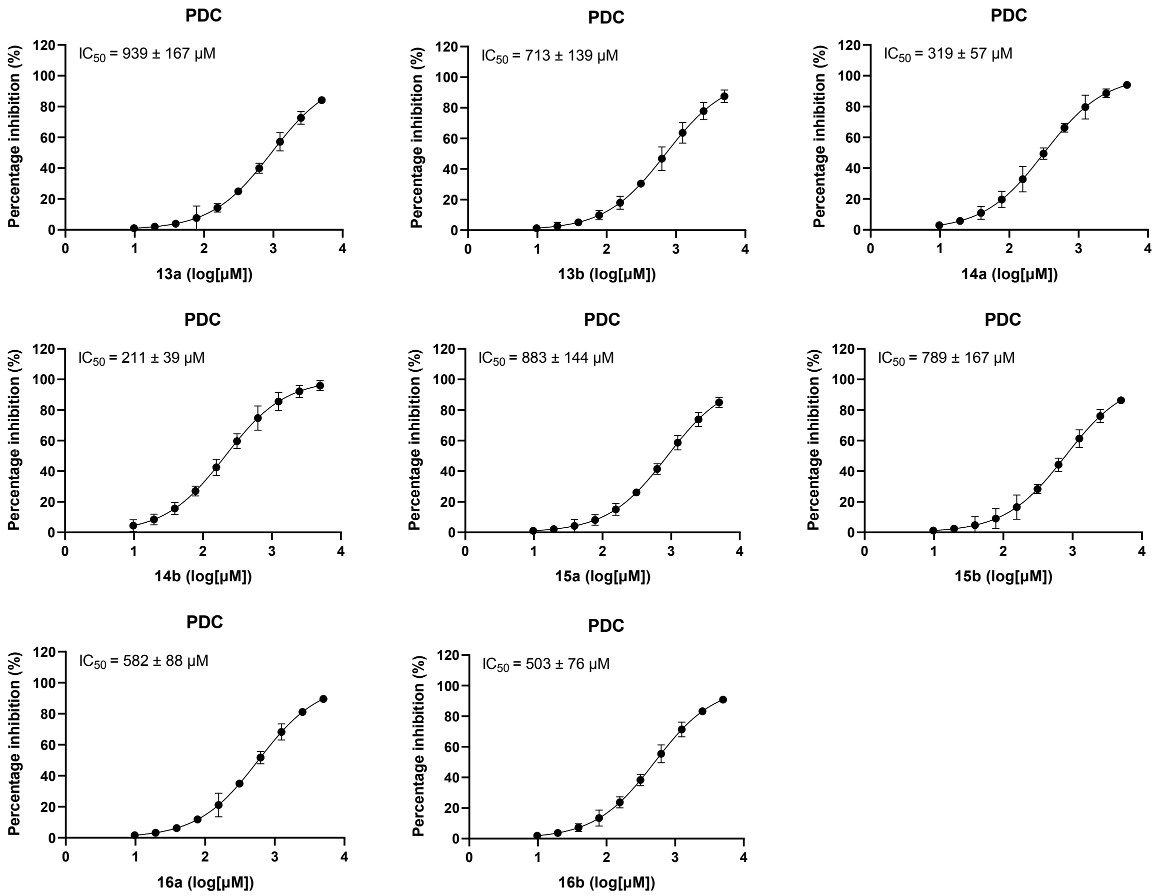

Figure S2. Measurement of *S. cerevisiae* PDC IC_{50} values at [TPP] = 150 μM. Measurements were made in triplicate. Where the error bars are not visible, they are smaller than the symbols. Best-fit nonlinear regression curves are shown.

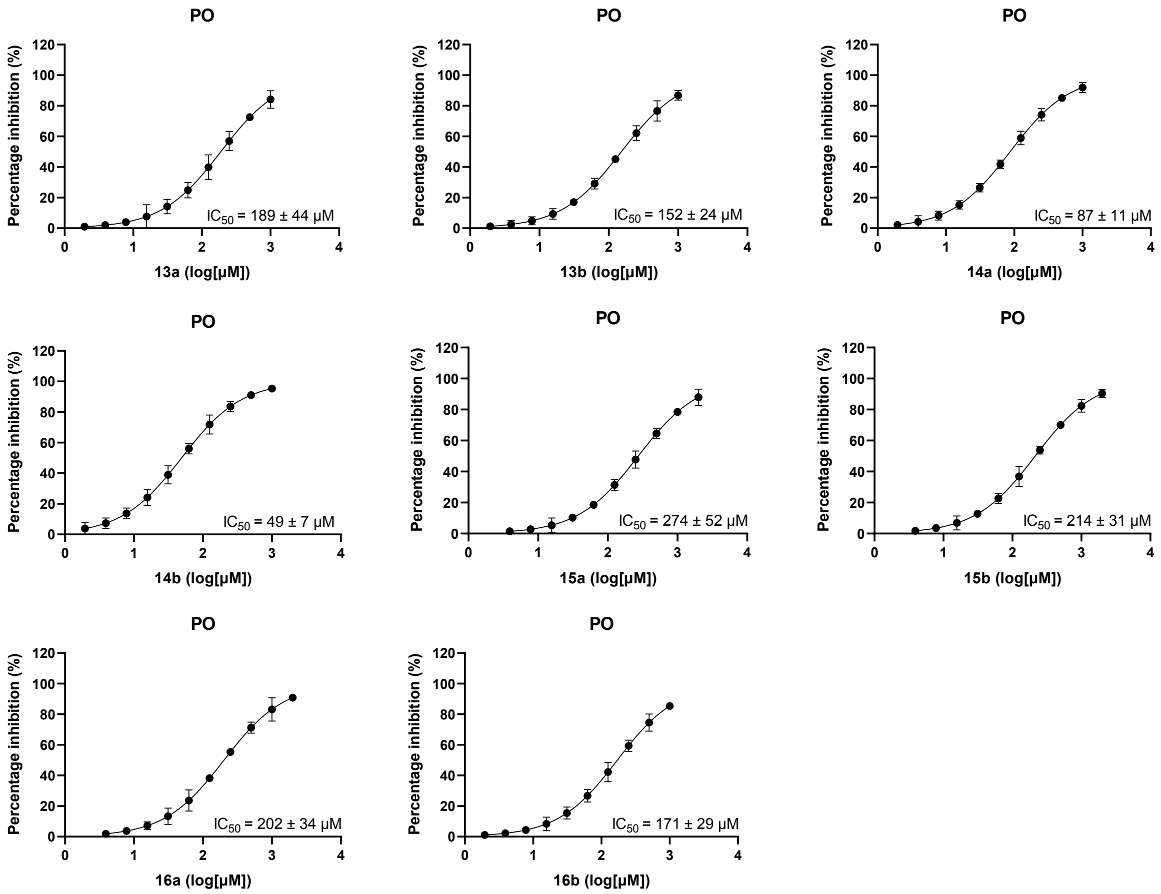

Figure S3. Measurement of *A. viridans* PO IC_{50} values at [TPP] = 60 µM. Measurements were made in triplicate. Where the error bars are not visible, they are smaller than the symbols. Best-fit nonlinear regression curves are shown.

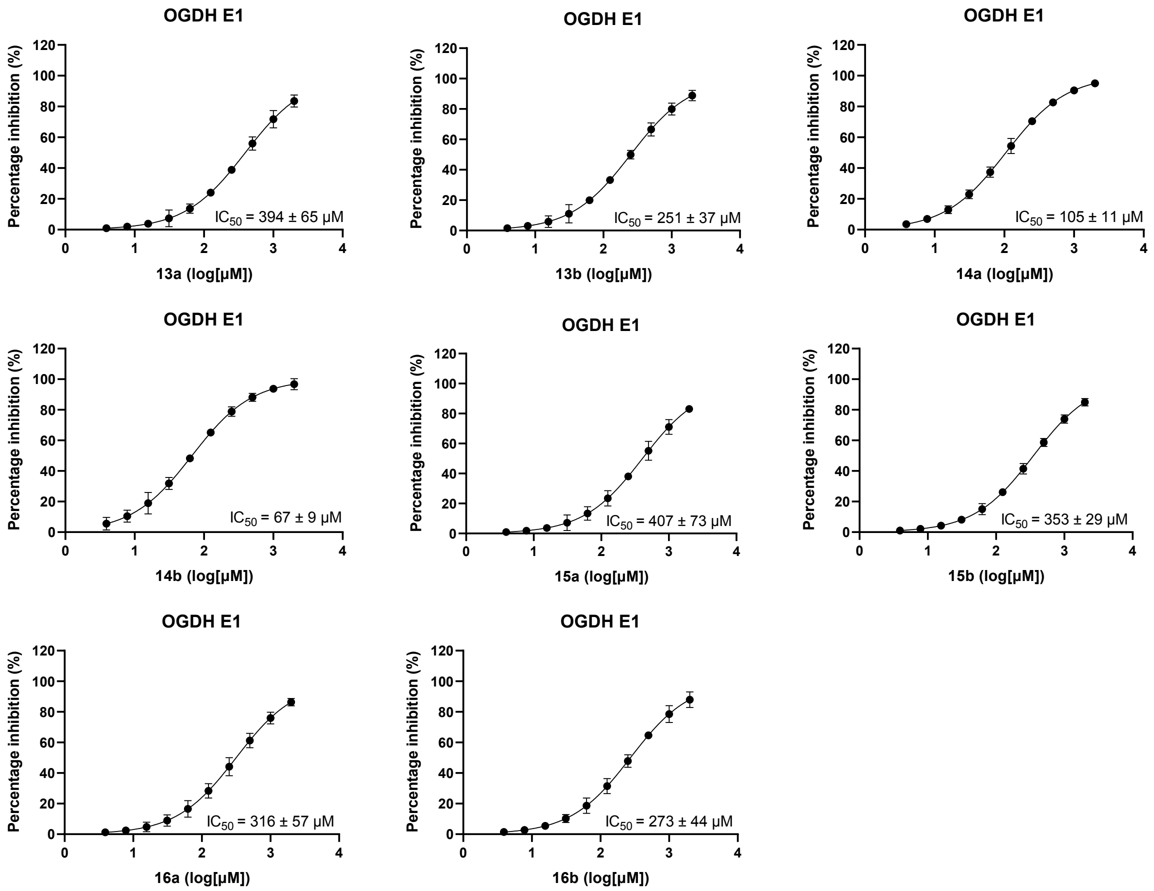

Figure S4. Measurement of *E. coli* OGDH E1 IC$_{50}$ values at [TPP] = 60 µM. Measurements were made in triplicate. Where the error bars are not visible, they are smaller than the symbols. Best-fit nonlinear regression curves are shown.

Table S1. Summary of inhibitory activity on TPP-dependent enzymes.[a]

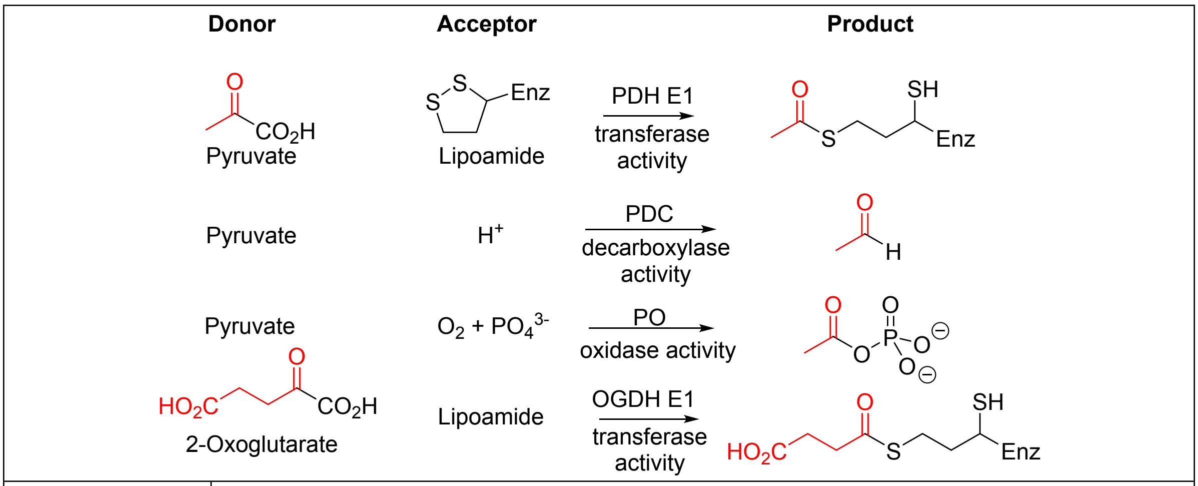

TPP-dependent enzymes	Compounds							
	Diesters		Dicarboxylates		Monoesters		Monocarboxylates	
	Short **13a**	Long **13b**	Short **14a**	Long **14b**	Short **15a**	Long **15b**	Short **16a**	Long **16b**
Porcine PDH E1								
% Inhibition (5:1)[b]	71 ± 3	81 ± 4	> 90	> 90	65 ± 4	71 ± 4	75 ± 4	77 ± 3
% Inhibition (1:1)[c]	36 ± 2	48 ± 3	62 ± 3	72 ± 3	25 ± 4	31 ± 3	34 ± 3	40 ± 3
IC_{50} (µM ± SEM)[d]	114 ± 15	68 ± 9	37 ± 6	25 ± 6	167 ± 35	126 ± 28	111 ± 18	92 ± 13
vs TPP[e]	0.53	0.88	1.62	2.40	0.36	0.48	0.54	0.65
K_I (µM)[f]	0.095	0.057	0.031	0.021	0.14	0.11	0.093	0.077
*Sc*PDC								
% Inhibition (5:1)[g]	40 ± 4	52 ± 3	70 ± 2	79 ± 3	42 ± 3	47 ± 4	55 ± 4	60 ± 4
% Inhibition (1:1)[h]	< 20	21 ± 5	31 ± 3	42 ± 3	< 20	< 20	24 ± 4	25 ± 5
IC_{50} (µM ± SEM)[d]	939 ± 167	713 ± 139	319 ± 57	211 ± 39	883 ± 144	789 ± 167	582 ± 88	503 ± 76
vs TPP[e]	0.16	0.21	0.47	0.71	0.17	0.19	0.26	0.30
K_I (µM)[f]	94	71	32	21	88	79	58	50
*Av*PO								
% Inhibition (5:1)[b]	62 ± 4	65 ± 5	76 ± 3	85 ± 4	52 ± 3	59 ± 3	59 ± 3	63 ± 3
% Inhibition (1:1)[c]	26 ± 4	29 ± 4	41 ± 3	55 ± 4	21 ± 4	24 ± 4	22 ± 5	28 ± 4
IC_{50} (µM ± SEM)[d]	189 ± 44	152 ± 24	87 ± 11	49 ± 7	274 ± 52	214 ± 31	202 ± 34	171 ± 29
vs TPP[e]	0.32	0.39	0.69	1.22	0.22	0.28	0.30	0.35
K_I (µM)[f]	16	13	7.3	4.1	23	18	17	14
*Ec*OGDH E1								
% Inhibition (5:1)[b]	40 ± 3	54 ± 3	76 ± 5	83 ± 4	38 ± 3	42 ± 4	49 ± 3	54 ± 4
% Inhibition (1:1)[c]	< 20	23 ± 4	36 ± 4	47 ± 3	< 20	< 20	< 20	22 ± 5
IC_{50} (µM ± SEM)[d]	394 ± 65	251 ± 37	105 ± 11	67 ± 9	407 ± 73	353 ± 29	316 ± 57	273 ± 44
vs TPP[e]	0.15	0.24	0.57	0.90	0.15	0.17	0.19	0.22
K_I (µM)[f]	20	13	5.3	3.4	20	18	16	14

[a] Data are the means of measurements in three technical replicates. [b] [Compound] = 250 µM, [TPP] = 50 µM. [c] [Compound] = [TPP] = 250 µM. [d] IC_{50} values determined at [TPP] = 60 µM (for PDH, PO and OGDH) and 150 µM (for PDC). [e] Affinity of the compound relative to that of TPP (= [TPP]/IC_{50}). [f] All compounds are TPP-competitive inhibitors so their K_I values are calculated according to the K_M values[7] for TPP, 0.05 µM (PDH), 15 µM (PDC), 5 µM (PO) and 3 µM (OGDH), using the equation [TPP]/IC_{50} = $K_{M(TPP)}/K_I$. [g] [Compound] = 750 µM, [TPP] = 150 µM. [h] [Compound] = [TPP] = 750 µM.

Computational docking – methods and results (Figure S5)

Docking of TPP and compounds was executed using CCDC GOLD docking program with PDB: 6CFO, 1VPD, 1V5F and 6U3J for human PDH E1, *Sc*PDC, *Av*PO and human OGDH E1, respectively. TPP or the equivalent ligands were selected as the binding site. Our molecules were generated using Mercury. GA runs were set at 50 and efficiency of docking calculations was set to "Very Flexible" (200%). No early termination was permitted. The active site was defined by ligands atoms of co-crystalised ligand A5X401 (PDB 6CFO). CHEMPLP and GoldScore were the docking scoring and rescoring respectively.[5] For all other GOLD-specific docking options the default settings were used. Interactions between docked compounds and protein models are shown using CCDC GOLD.

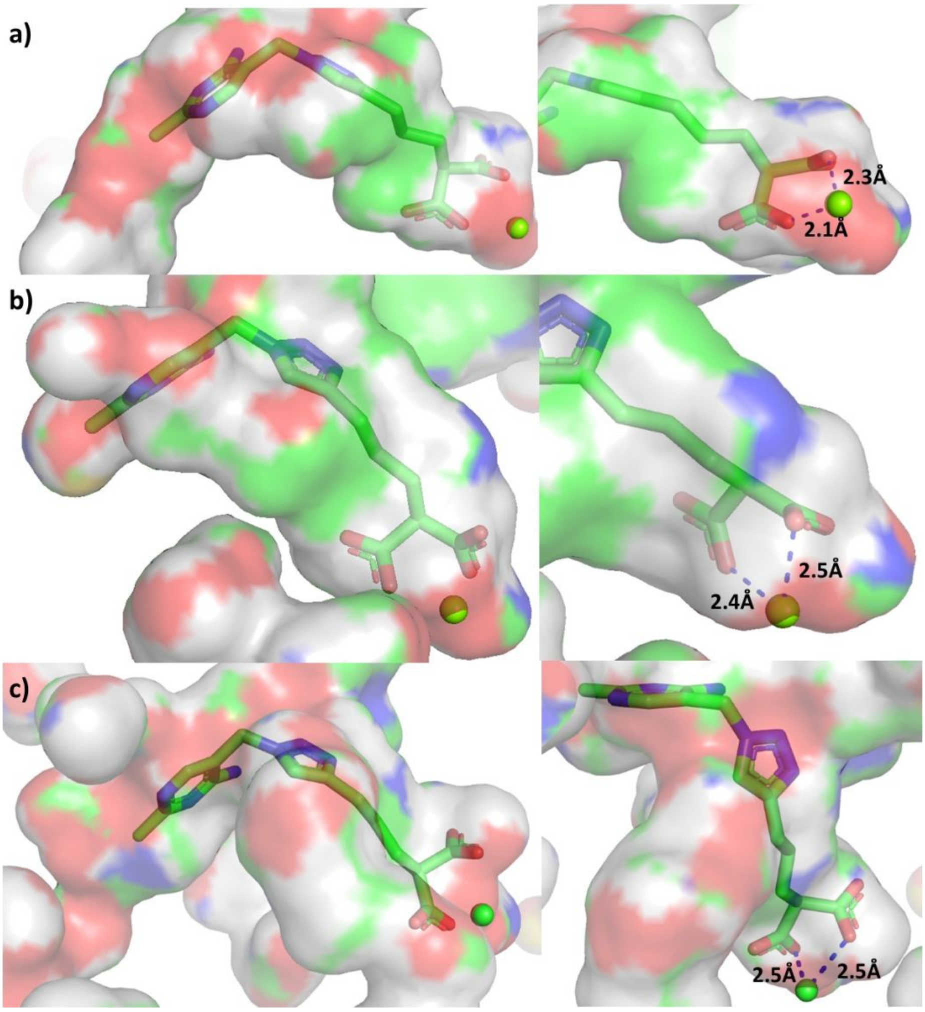

Figure S5. Left column: Predicted binding modes of **14b** (green carbon atoms) in the active site (shown as surface) of **a)** *Sc*PDC (1PVD), **b)** *Av*PO (1V5F), and **c)** human OGDH E1 (6U3J). The binding modes of the competitive inhibitors are similar to that of TPP – all displaying a V-shaped conformation between aminopyrimidine and the thiazolium ring. **Right column:** Views showing the interactions between the MBG and the Mg^{2+} (represented as a yellowish-green sphere).

PAMPA – methods and results (Table S2)

PAMPA was carried out in 96-well microtiter filter plates obtained from Millipore as described[1] with some modifications. Each well of the filter plate was impregnated with 15 µL of 5% hexadecane dissolved in hexane (*i.e.* total amount of hexadecane: 0.75 µL) for at least 10 minutes in ventilated environment to allow for complete evaporation of hexane. Donor compartments were filled with 200 µL compound-containing donor solutions of compounds dissolved in 5% DMSO, phosphate buffered saline PBS) and connected to acceptor plate prefilled with buffer (5% DMSO in PBS, pH 7.4). The resulting sandwich was incubated at room temperature under gentle shaking and wrapped in wet paper towels to avoid evaporation. After 10 hours, the sandwich was disassembled and the solutions in the acceptor and donor were transferred to a disposable UV-transparent plate. UV absorption was measured at wavelengths between 220 and 340 nm using a microplate reader (CLARIOstar). Compounds were tested at 500 µM. Calibration to determine concentration of compounds were performed with varying compound concentrations in buffer (5% DMSO in PBS).

The artificial membrane permeability is expressed as fraction absorbed (Fa%)[2] or log P_e (log of the effective permeability)[1]. LogD values at pH 7.4 were calculated using MarvinSketch.

$$Fa\% = 100 \cdot C_A \cdot V_A / C_{D0} \cdot V_D$$

where:
C_A = final drug concentration in the acceptor well (µM)
V_A = volume in the acceptor well (cm^3)
C_{D0} = Initial drug concentration in the donor well (µM)
V_D = volume in the donor well (cm^3)

$$\log P_e \text{ (cm/s)} = \log\left[\frac{-\ln\left[1 - C_A/C_{equilibrium}\right]}{S(1/V_D + 1/V_A)t}\right]$$

where:
C_A = final drug concentration in the acceptor well (µM)
V_A = volume in the acceptor well (cm^3)
V_D = volume in the donor well (cm^3)
S = surface area (cm^2), typically 0.268 cm^2
t = incubation time(s)
$C_{equilibrium}$ = theoretical equilibrium concentration = $[C_D \cdot V_D + C_A \cdot V_A] / [V_D + V_A]$
where:
C_D = final drug concentration in the donor well (µM)
V_D = volume in the donor well (cm^3)
C_A = final drug concentration in the acceptor well (µM)
V_A = volume in the acceptor well (cm^3)

Table S2. Permeability data.

Compounds	Fa (%)	PAMPA log P_e (cm/s)	cLogD (pH = 7.4)
**5**	2	-6.4	-3.8
**12**	48	-5.1	+3.9
**13a**	39	-5.2	+0.4
**13b**	42	-5.2	+0.8
**14a**	2	-6.5	-5.5
**14b**	1	-6.8	-4.5
**15a**	38	-5.3	+0.5
**15b**	44	-5.2	+0.9
**16a**	3	-6.4	-2.9
**16b**	2	-6.4	-2.3

UPLC-HRMS analysis of the esterase treatment – methods and results

Metabolic stability against esterase was conducted as described[6] with some modifications. Porcine liver esterase (PLE) was purchased from Sigma. UPLC was performed on a Waters' Acquity UPLC H-Class equipped with a C18 reverse-phase column. Separation was achieved with a flowrate of 0.4 mL/min and the following mobile phase: 0.1% formic acid in H_2O (A) and 0.1% formic acid in acetonitrile (B). Starting with 90% A and 10% B, an isocratic gradient was run for 7 min to a final solvent mixture of 100% B, which was held for 30 s before ramping back down to 90% A and 10% B over 1 min. Compounds were prepared in HEPES buffer (50 mM, pH 7.4) at a concentration of 0.2 mM. Retention times of compounds were determined under identical UPLC conditions prior to evaluation of esterase cleavage of the protected compound.

To determine the efficiency of esterase cleavage for the compounds, a 0.2 mM solution of test compound (1 mL) in HEPES buffer (50 mM, pH 7.4) was prepared and treated with PLE (15 U). The sample was incubated at 25 ^{o}C for 2 h or 24 h prior to analysis.

Synthetic Procedures

General synthesis methods

Oxygen- and moisture-sensitive reactions were carried out in flame-dried glassware under a nitrogen atmosphere. Unless otherwise stated, all chemicals and reagents were purchased from commercial suppliers and used without further purification.

Reaction progress was monitored by analytical thin-layer chromatography (TLC). TLC was conducted using Merck glass plates with silica Kieselgel 60 F254 of thickness 0.25 mm and visualised under 254 nm UV lamp or potassium permanganate staining solution (with light heating).

Flash column chromatography was carried out in the indicated solvent system using prepacked silica gel cartridges for use on the Biotage Purification System.

All solvents were removed under reduced pressure using a Büchi rotary evaporator with dry ice traps.

All yields refer to chromatographically and spectroscopically pure compounds unless otherwise stated. Compounds were characterised by, at minimum, ^{1}H NMR spectroscopy, ^{13}C NMR spectroscopy and HRMS, unless otherwise stated.

Melting points of compounds were measured using a Reichert machine and are uncorrected.

^{1}H NMR spectra were recorded at 400 MHz in $CDCl_3$, CD_3OD, D_2O, or CD_3SOCD_3 solution on a Bruker 400 MHz or 700 MHz spectrometer and chemical shifts were recorded in parts per million (ppm). ^{13}C NMR spectra were recorded on a Bruker 400 MHz or 700 MHz spectrometer. Resonances are described using the following abbreviations: s (singlet), d (doublet), t (triplet), q (quartet), qnt (quintet), sext (sextet), m (multiplet), br (broad), dd (doublet of doublets), *etc*. Coupling constants (*J*) are given in Hz and are rounded to the nearest 0.1 Hz. All NMR data were collected at 25 °C.

Mass spectra used electrospray ionisation (ESI).

Experimental procedures (Synthesis)

5-(Azidomethyl)-2-methylpyrimidin-4-amine **6**

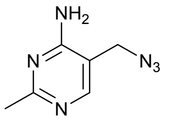

To a stirred solution of thiamine hydrochloride (10.0 g, 29.7 mmol) and NaN_3 (5.0 g, 76.9 mmol) in water (90 mL, 0.33 M) was added Na_2SO_3 (0.4 g, 3.2 mmol). The resultant mixture was stirred at 70 °C overnight, then acidified with citric acid monohydrate (10.5 g) to pH 4-5, washed with DCM (200 mL), and basified with potassium carbonate to pH 8-10. Upon product precipitation, the suspension was filtered under reduced pressure. The residue was rinsed with cold water and dried under reduced pressure to yield azide **6** as a white solid (3.3 g, 68% yield), which was used in the next step without further purification. **^{1}H NMR** (400 MHz, CD_3OD) δ 8.01 (s, 1H), 4.33 (s, 2H), 2.43 (s, 3H). Analytical data are consistent with those previously reported.[7]
Note: this work-up procedure gave a higher yield than previously reported.

2-Ethylbutyl N-((2-(1-((4-amino-2-methylpyrimidin-5-yl)methyl)-1H-1,2,3-triazol-4-yl)ethoxy)(phenoxy)phosphoryl)-L-alaninate **12**

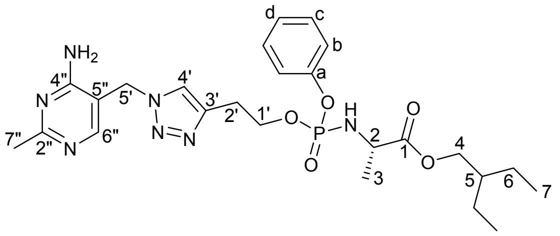

To a stirred solution of alaninate[8] **11** (170 mg, 0.38 mmol) and $MgCl_2$ (29 mg, 0.31 mmol) in dry MeCN (1.0 mL, 0.3 M) under nitrogen at r.t. was added alcohol **7a** (74 mg, 0.31 mmol). The resultant mixture was stirred at 50 °C for 10 min, treated with DIEPA (0.13 mL, 0.78 mmol), stirred at 50 °C overnight, diluted with EtOAc (20 mL), washed with aqueous phosphate buffer (pH 7) (100 mL), dried over $MgSO_4$, filtered, and evaporated under reduced pressure. The residue was purified by silica flash chromatography (5% MeOH in DCM) to yield triazole **12** (a diastereomeric mixture at the P atom) as a white semi-solid (79 mg, 47%). **^{1}H NMR** (400 MHz, $CDCl_3$) δ 8.16 (s, 1H, H-6”), 7.28-7.34 (m, 3H, H-4’ and H-c), 7.14-7.18 (m, 3H, H-b and H-d), 5.76 (br, 2H, NH_2), 5.28 (s, 2H, H-5’), 4.33-4.37 (m, 2H, H-1’), 3.93-4.07 (m, 3H, H-2 and H-4), 3.76-3.82 (m, 1H, NH), 3.09 (q, 2H, *J* = 6.6 Hz, H-2’), 2.48 (s, 3H, H-7”), 1.49-1.52 (m, 1H, H-5), 1.31-1.37 (m, 7H, H-3 and H-6), 0.87 (t, 6H, *J* = 7.5 Hz, H-7). **^{13}C NMR** (100 MHz, $CDCl_3$) δ 173.6 (C-1), 168.8 (C-2”), 161.9 (C-4”), 156.0 (C-6”), 150.7 (C-a), 144.7 (C-3’), 129.6 (C-c), 124.9 (C-d), 122.1 (C-4’), 120.2 (C-b), 108.0 (C-5”), 67.6 (C-4), 65.5 (C-1’), 50.3 (C-2), 48.6 (C-5’), 40.2 (C-5), 27.1 (C-2’), 25.6 (C-7”), 23.1 (C-6), 21.0 (C-3), 10.9 (C-7). **^{31}P NMR** (162 MHz, $CDCl_3$) δ 2.37 and 2.16. **HRMS** (ESI) *m/z*: [M+H$^+$] calculated for $C_{25}H_{36}N_7O_5P$: 546.2593; found: 546.2599.

General procedure for preparation of diesters 13a and 13b:

To a stirred solution of dimethyl malonate (0.24 mL, 2.0 mmol) in dry DMF (1.0 mL, 0.2 M) under nitrogen at 0 °C was added NaH (60% in mineral oil, 80 mg, 2.0 mmol). The resultant mixture was stirred at r.t. for 1 h, treated with tosylate[7] **8** (1.0 mmol), stirred at 40 °C for 3 days, quenched with aqueous phosphate buffer (pH 7) (30 mL), and extracted with *n*-BuOH (3 x 50 mL). The combined organic phases were dried over $MgSO_4$, filtered, and evaporated under reduced pressure. The residue was purified by silica flash chromatography (5-15% MeOH in DCM) to yield diester **13**.

Dimethyl 2-(2-{1-[(4-amino-2-methylpyrimidin-5-yl)methyl]-1H-1,2,3-triazol-4-yl}ethyl)propanedioate **13a**

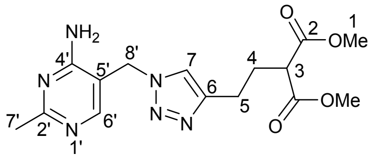

Prepared from tosylate **8a**. White semi-solid (226 mg, 65%). **^{1}H NMR** (400 MHz, CD_3OD) δ 8.06 (s, 1H, H-6'), 7.81 (s, 1H, H-7), 5.47 (s, 2H, H-8'), 3.70 (s, 6H, H-1), 3.48 (t, 1H, *J* = 7.3 Hz, H-3), 2.75 (t, 2H, *J* = 7.8 Hz, H-5), 2.42 (s, 3H, H-7'), 2.22 (app q., 2H, *J* = 7.5 Hz, H-4). **^{13}C NMR** (100 MHz, CD_3OD) δ 169.6 (C-2), 167.4 (C-4'), 162.1 (C-2'), 154.7 (C-6'), 146.7 (C-6), 122.1 (C-7), 108.4 (C-5'), 51.5 (C-1), 50.4 (C-3), 47.1 (C-8'), 28.0 (C-4), 23.5 (C-7'), 22.5 (C-5). **HRMS** (ESI) *m/z*: [M+H$^+$] calculated for $C_{15}H_{20}N_6O_4$: 349.1618; found: 349.1622.

Dimethyl 2-(3-{1-[(4-amino-2-methylpyrimidin-5-yl)methyl]-1H-1,2,3-triazol-4-yl}propyl)propanedioate **13b**

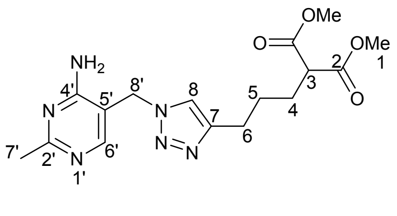

Prepared from tosylate **8b**. Colourless oil (250 mg, 69%). **^{1}H NMR** (400 MHz, CD_3OD) δ 8.06 (s, 1H, H-6'), 7.79 (s, 1H, H-8), 5.47 (s, 2H, H-8'), 3.70 (s, 6H, H-1), 2.70-2.75 (m, 3H, H-3 and H-6), 2.42 (s, 3H, H-7'), 1.86-1.94 (m, 2H) and 1.64-1.72 (m, 2H) (H-4 and H-5). **^{13}C NMR** (100 MHz, CD_3OD) δ 169.8 (C-2), 167.5 (C-4'), 162.1 (C-2'), 155.0 (C-6'), 147.6 (C-7), 121.9 (C-8), 108.5 (C-5'), 51.5 (C-1), 50.9 (C-3), 47.1 (C-8'), 27.8, 26.5, 24.4 (C-4, C-5 and C-6), 23.6 (C-7'). **HRMS** (ESI) *m/z*: [M+H$^+$] calculated for $C_{16}H_{22}N_6O_4$: 363.1774; found: 363.1783.

General procedure for preparation of monoesters 15a and 15b:

To a stirred solution of diester **13** (0.6 mmol) in dry DMSO (3.0 mL, 0.2 M) at r.t. was added water (0.06 mL, 3.0 mmol) and LiCl (252 mg, 6.0 mmol). The resultant mixture was stirred at 125 ^{o}C overnight, diluted with *n*-BuOH (70 mL), washed with water (30 mL), dried over $MgSO_4$, filtered, and evaporated under reduced pressure. The residue was purified by silica flash chromatography (5-15% MeOH in DCM) to yield monoester **15** as a solid.

Methyl 4-{1-[(4-amino-2-methylpyrimidin-5-yl)methyl]-1H-1,2,3-triazol-4-yl}butanoate **15a**

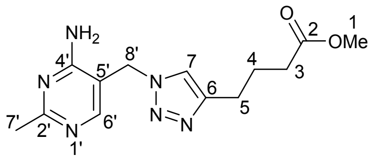

Prepared from diester **13a**. Pale yellow semi-solid (70 mg, 40%). **^{1}H NMR** (400 MHz, CD_3OD) δ 8.05 (s, 1H, H-6'), 7.80 (s, 1H, H-7), 5.47 (s, 2H, H-8'), 3.65 (s, 3H, H-1), 2.74 (t, 2H, *J* = 7.7 Hz, H-5), 2.42 (s, 3H, H-7'), 2.37 (t, 2H, *J* = 7.7 Hz, H-3), 1.96 (qnt, 2H, *J* = 7.7 Hz, H-4). **^{13}C NMR** (100 MHz, CD_3OD) δ 173.9 (C-2), 167.2 (C-4'), 162.2 (C-2'), 154.3 (C-6'), 147.4 (C-6), 122.1 (C-7), 108.6 (C-5'), 50.6 (C-1), 47.1 (C-8'), 32.5 (C-3), 24.2 and 24.1 (C-4 and C-5), 23.4 (C-7'). **HRMS** (ESI) *m/z*: [M+H$^+$] calculated for $C_{13}H_{18}N_6O_2$: 291.1563; found: 291.1569.

Methyl 5-{1-[(4-amino-2-methylpyrimidin-5-yl)methyl]-1H-1,2,3-triazol-4-yl}pentanoate **15b**

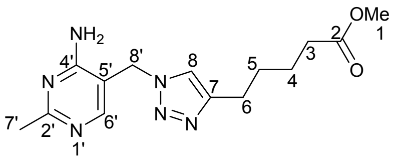

Prepared from diester **13b**. yellow oil (82 mg, 45%). ^{1}**H NMR** (400 MHz, CD_3OD) δ 8.04 (s, 1H, H-6’), 7.79 (s, 1H, H-8), 5.47 (s, 2H, H-8’), 3.65 (s, 3H, H-1), 2.71 (t, 2H, *J* = 7.1 Hz, H-6), 2.42 (s, 3H, H-7’), 2.35 (t, 2H, *J* = 6.9 Hz, H-3), 1.61-1.72 (m, 4H, H-4 and H-5). ^{13}**C NMR** (100 MHz, CD_3OD) δ 174.3 (C-2), 167.5 (C-4’), 162.2 (C-2’), 154.9 (C-6’), 148.0 (C-7), 121.9 (C-8), 108.6 (C-5’), 50.5 (C-1), 47.1 (C-8’), 32.9 (C-3), 28.4, 24.5, 23.9 (C-4, C-5 and C-6), 23.6 (C-7’). **HRMS** (ESI) *m/z*: [M+H$^+$] calculated for $C_{14}H_{20}N_6O_2$: 305.1720; found: 305.1733.

General procedure for preparation of carboxylates 14a, 14b, 16a and 16b:

To a stirred solution of corresponding ester (0.2 mmol) in THF (0.5 mL) and water (0.5 mL) at r.t. was added 1 M aq. KOH (0.2 mL for **15** or 0.4 mL for **13**). The resultant mixture was stirred at 40 °C for 4 h and concentrated under reduced pressure to yield carboxylates **14** and **16** as solids.

Dipotassium 2-(2-{1-[(4-amino-2-methylpyrimidin-5-yl)methyl]-1H-1,2,3-triazol-4-yl}ethyl)propanedioate **14a**

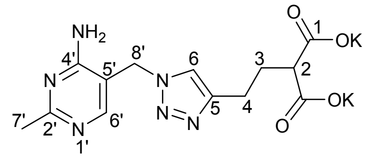

Prepared from diester **13a**. White solid (76 mg, 96%). **m.p.** 218-221 °C. ^{1}**H NMR** (400 MHz, D_2O) δ 7.95 (s, 1H, H-6’), 7.69 (s, 1H, H-6), 5.34 (s, 2H, H-8’), 2.95 (t, 1H, *J* = 7.7 Hz, H-2), 2.57 (t, 2H, *J* = 7.7 Hz, H-4), 2.30 (s, 3H, H-7’), 1.93 (app q., 2H, *J* = 7.7 Hz, H-3). ^{13}**C NMR** (100 MHz, D_2O) δ 179.1 (C-1), 167.7 (C-4’), 161.6 (C-2’), 155.5 (C-6’), 148.2 (C-5), 123.1 (C-6), 108.3 (C-5’), 57.8 (C-2), 47.5 (C-8’), 29.8 (C-3), 23.8 (C-7’), 23.3 (C-4). **HRMS** (ESI) *m/z*: [M+H$^+$] calculated for $C_{13}H_{16}N_6O_4$: 321.1305; found: 321.1316.

Dipotassium 2-(3-{1-[(4-amino-2-methylpyrimidin-5-yl)methyl]-1H-1,2,3-triazol-4-yl}propyl)propanedioate **14b**

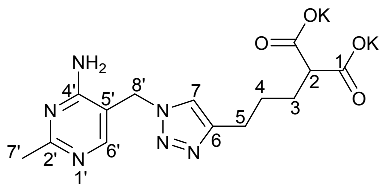

Prepared from diester **13b**. White solid (80 mg, 98%). **m.p.** 222-225 °C. ^{1}**H NMR** (400 MHz, D_2O) δ 7.97 (s, 1H, H-6’), 7.69 (s, 1H, H-7), 5.35 (s, 2H, H-8’), 2.98 (t, 1H, *J* = 8.1 Hz, H-2), 2.62 (t, 2H, *J* = 7.1 Hz, H-5), 2.32 (s, 3H, H-7’), 1.58-1.66 (m, 2H) and 1.46-1.55 (m, 2H) (H-3 and H-4). ^{13}**C NMR** (100 MHz, D_2O) δ 179.6 (C-1), 167.8 (C-4’), 161.7 (C-2’), 155.5 (C-6’), 148.8 (C-6), 123.0 (C-7), 108.3 (C-5’), 58.4 (C-2), 47.5 (C-8’), 29.6, 27.2 and 24.4 (C-3, C-4 and C-5), 23.9 (C-7’). **HRMS** (ESI) *m/z*: [M+H$^+$] calculated for $C_{14}H_{18}N_6O_4$: 335.1461; found: 335.1480.

Potassium 4-{1-[(4-amino-2-methylpyrimidin-5-yl)methyl]-1H-1,2,3-triazol-4-yl}butanoate **16a**

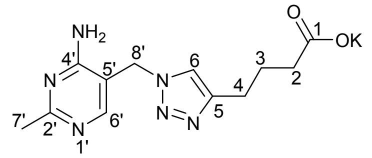

Prepared from monoester **15a**. White solid (60 mg, 96%). **m.p.** 185-187 °C. **^{1}H NMR** (400 MHz, CD_3OD) δ 8.06 (s, 1H, H-6'), 7.81 (s, 1H, H-6), 5.46 (s, 2H, H-8'), 2.73 (t, 2H, J = 7.7 Hz, H-2), 2.42 (s, 3H, H-7'), 2.20 (t, 2H, J = 7.6 Hz, H-4), 1.93 (qnt, 2H, J = 7.6 Hz, H-3). **^{13}C NMR** (100 MHz, CD_3OD) δ 180.8 (C-1), 167.5 (C-4'), 162.1 (C-2'), 155.0 (C-6'), 148.2 (C-5), 122.0 (C-6), 108.6 (C-5'), 47.1 (C-8'), 37.0 (C-2), 26.1, 24.8 (C-3 and C-4), 23.6 (C-7'). **HRMS** (ESI) *m/z*: [M+H$^+$] calculated for $C_{12}H_{16}N_6O_2$: 277.1407; found: 277.1418.

Potassium 5-{1-[(4-amino-2-methylpyrimidin-5-yl)methyl]-1H-1,2,3-triazol-4-yl}pentanoate **16b**

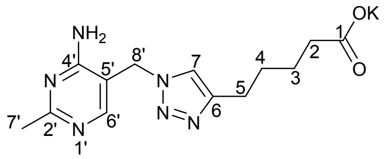

Prepared from monoester **15b**. White solid (65 mg, 99%). **m.p.** 189-192 °C. **^{1}H NMR** (400 MHz, D_2O) δ 7.94 (s, 1H, H-6'), 7.64 (s, 1H, H-7), 5.32 (s, 2H, H-8'), 2.56 (t, 2H, J = 7.3 Hz, H-5), 2.30 (s, 3H, H-7'), 2.07 (t, 2H, J = 7.0 Hz, H-2), 1.39-1.53 (m, 4H, H-3 and H-4). **^{13}C NMR** (100 MHz, D_2O) δ 183.6 (C-1), 167.7 (C-4'), 161.6 (C-2'), 155.5 (C-6'), 148.8 (C-6), 122.9 (C-7), 108.3 (C-5'), 47.5 (C-8'), 37.2(C-2), 28.3, 25.2, 24.2 (C-3, C-4 and C-5), 23.8 (C-7'). **HRMS** (ESI) *m/z*: [M+H$^+$] calculated for $C_{13}H_{18}N_6O_2$: 291.1563; found: 291.1580.

NMR spectra

^{1}H NMR of **12** in $CDCl_3$:

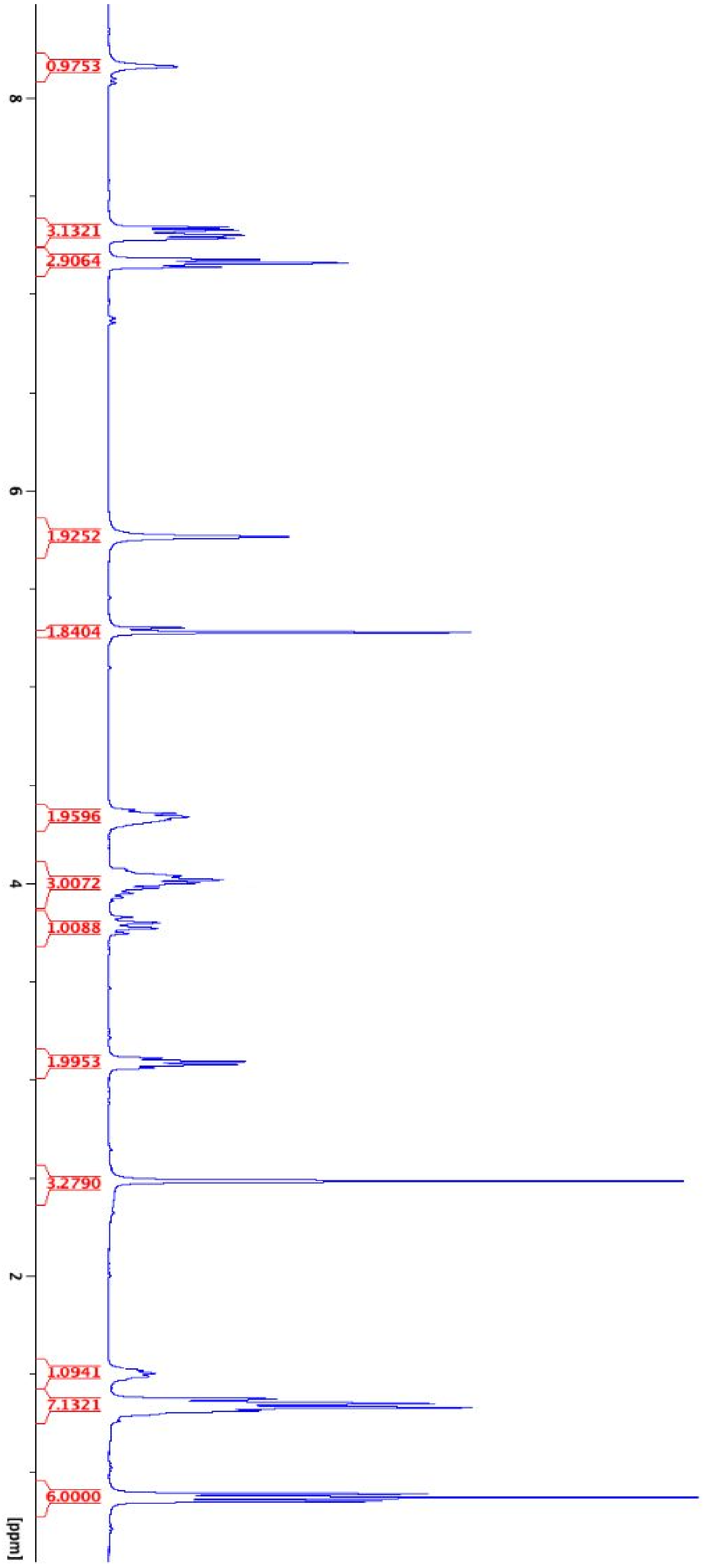

^{13}C NMR of **12** in $CDCl_3$:

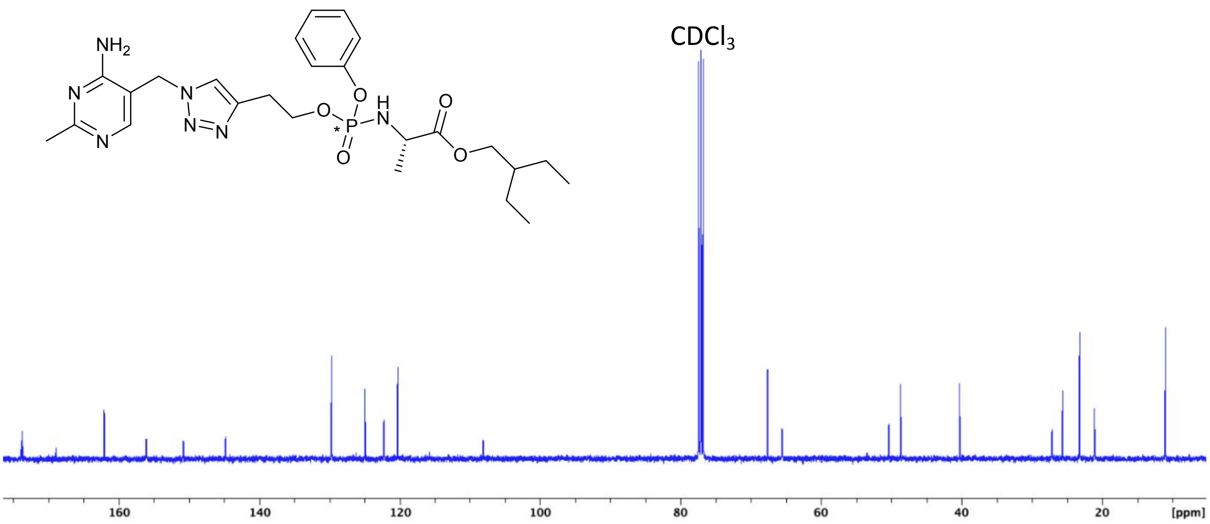

^{31}P NMR of **6a** in $CDCl_3$:

Although the two diastereomers are almost fully identical in both the ^{1}H and ^{13}C spectra, the presence of two peaks in the ^{31}P spectrum provides the evidence that the sample is indeed a diastereomeric mixture, not a single enantiomer.

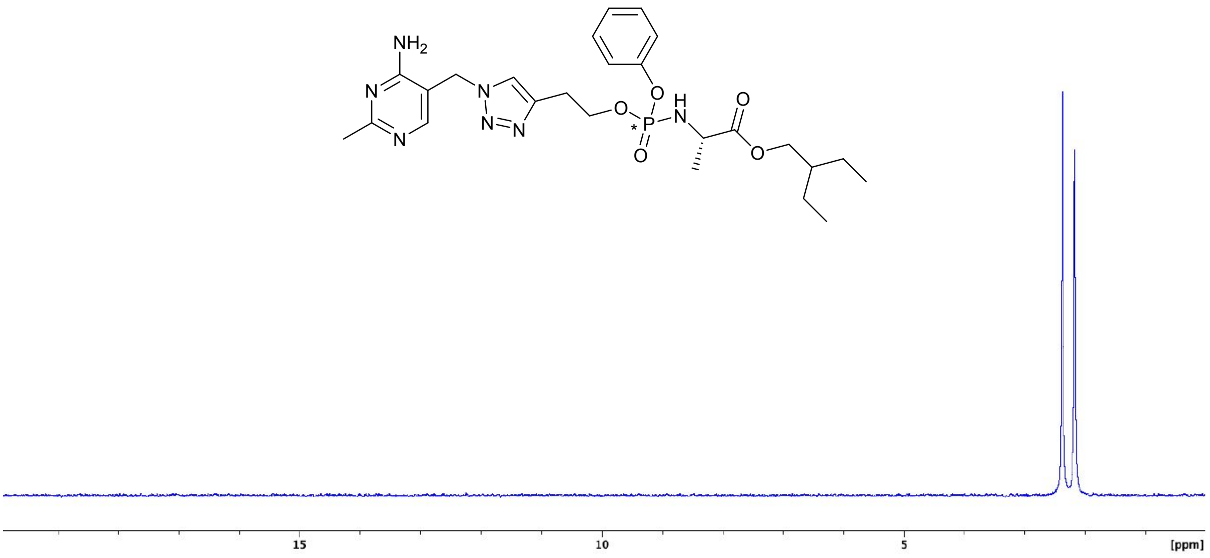

^{1}H NMR of **13a** in CD_3OD:

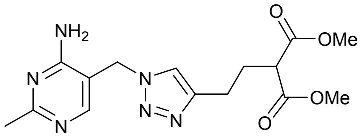

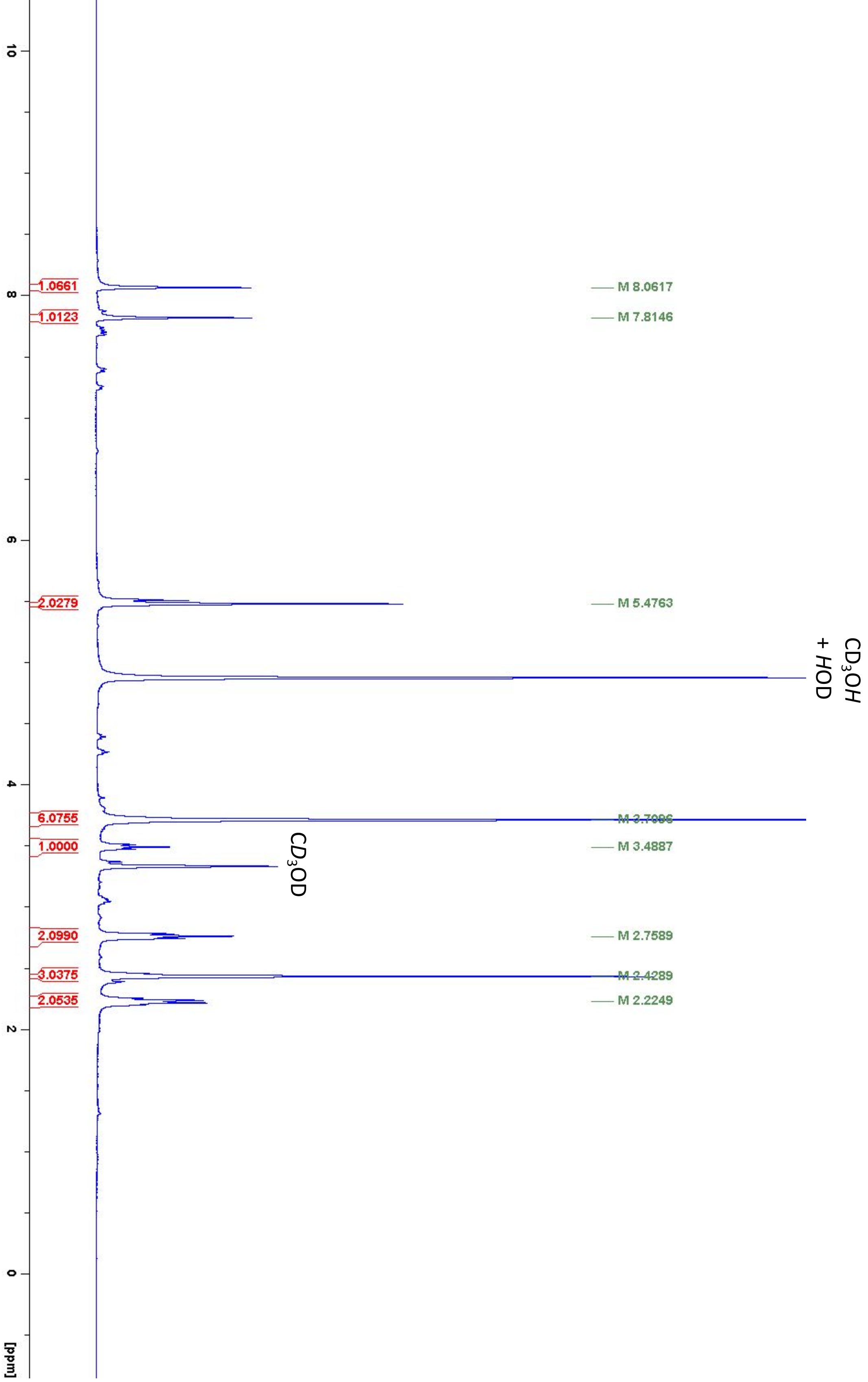

^{13}C NMR of **13a** in CD_3OD:

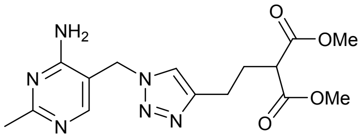

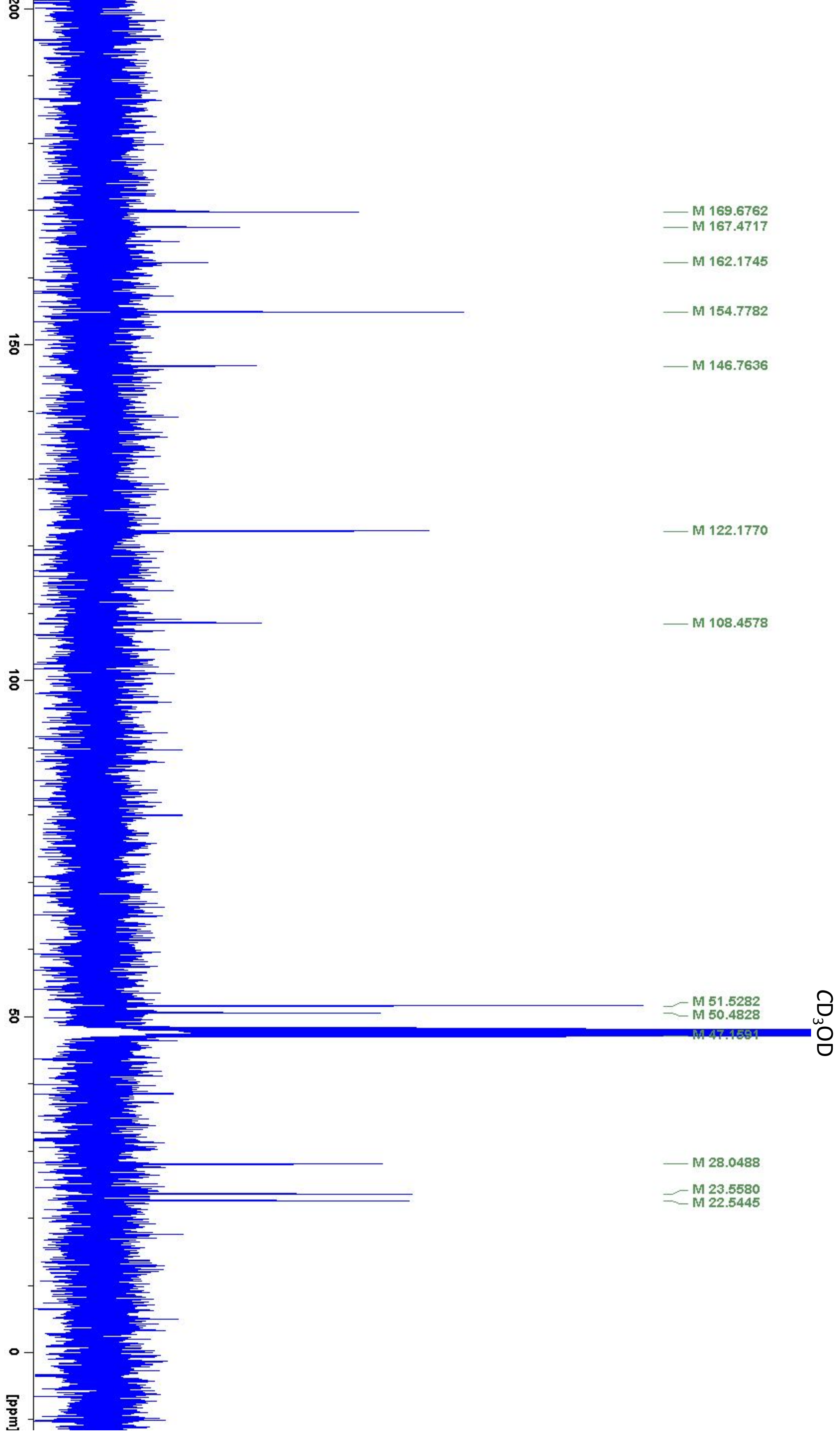

^{1}H NMR of **13b** in CD_3OD:

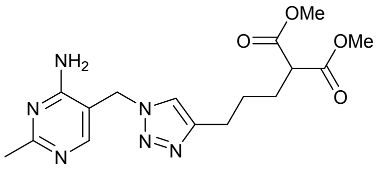

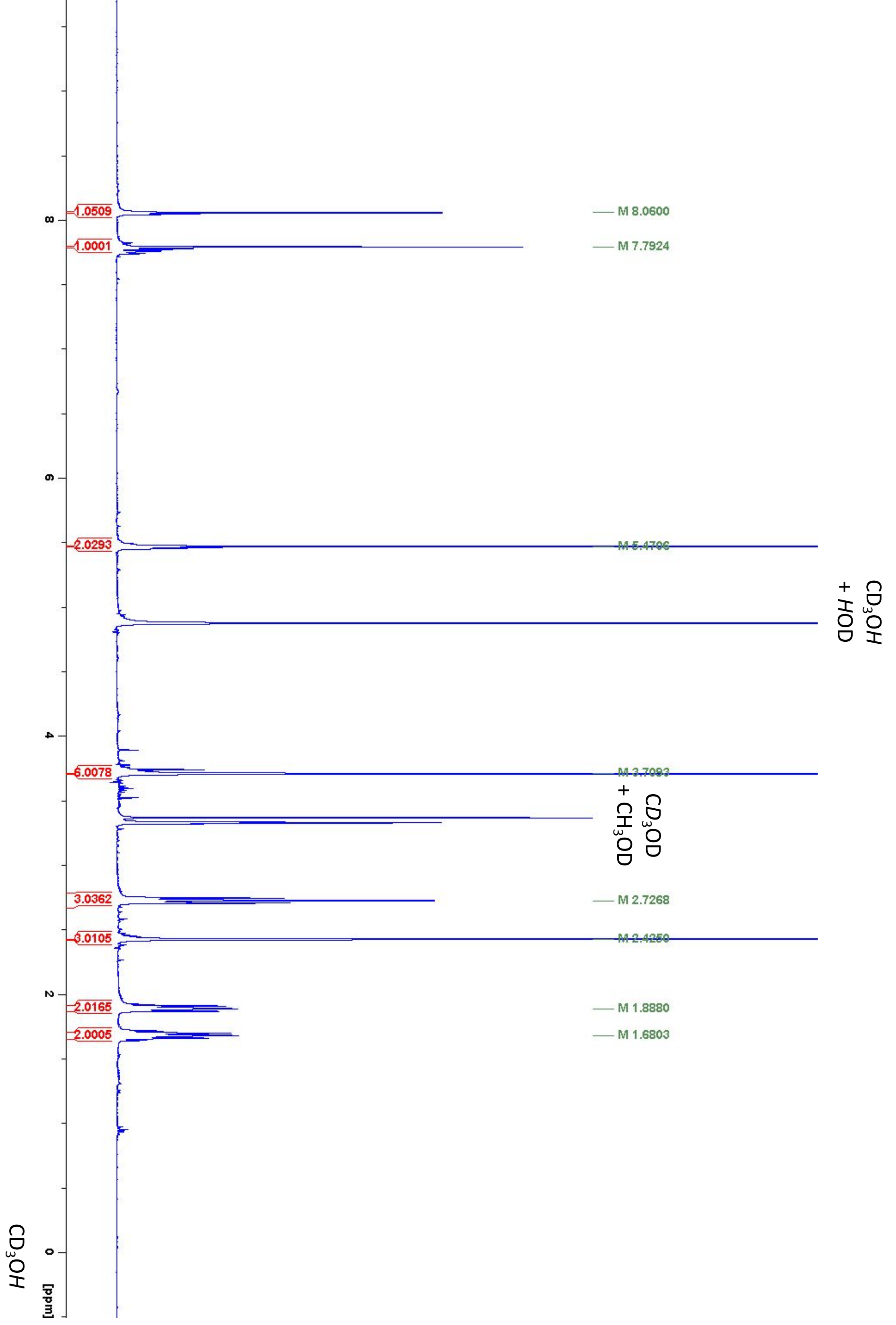

^{13}C NMR of **13b** in CD_3OD:

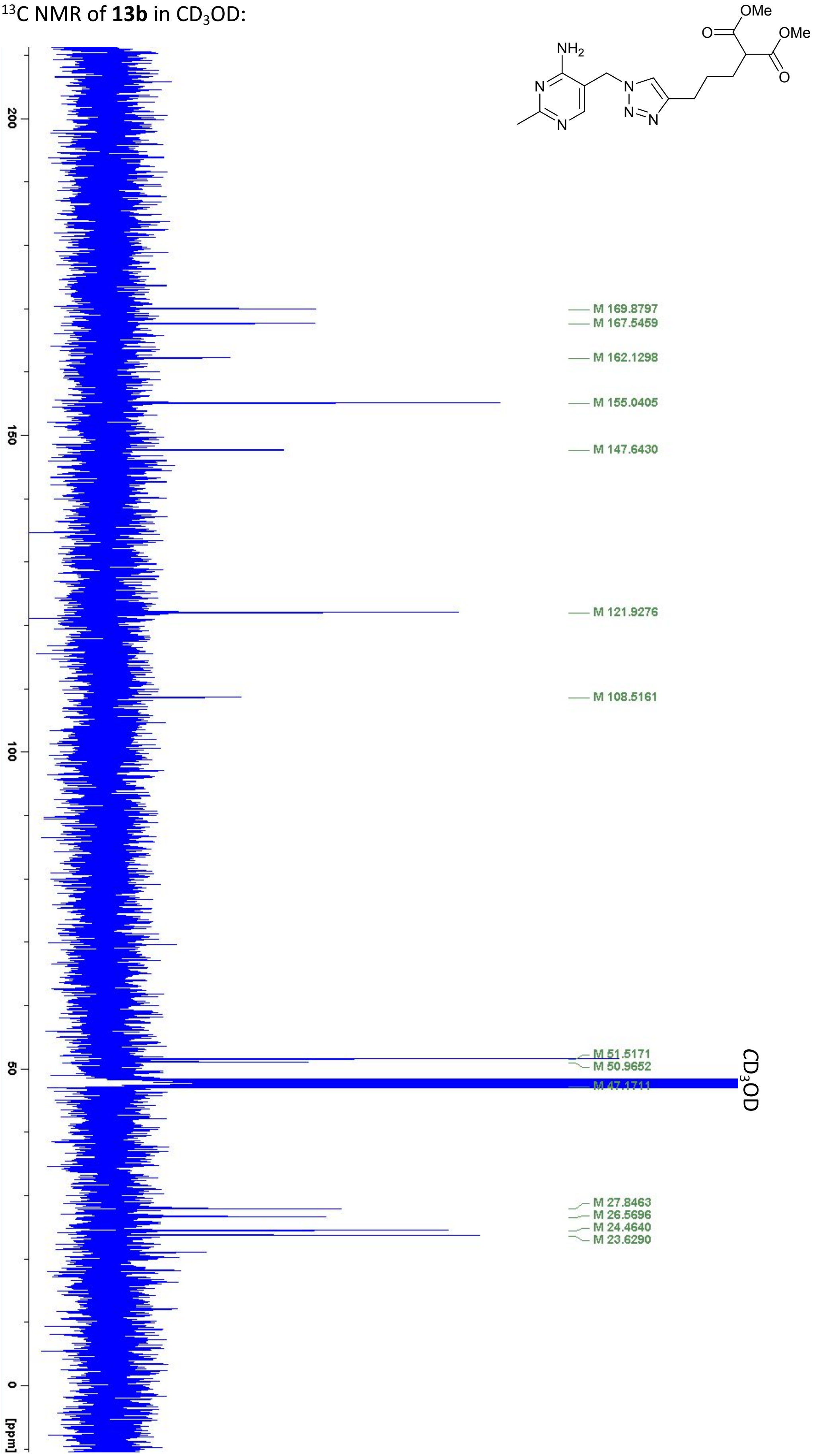

^1H NMR of **14a** in D_2O:

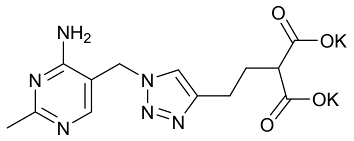

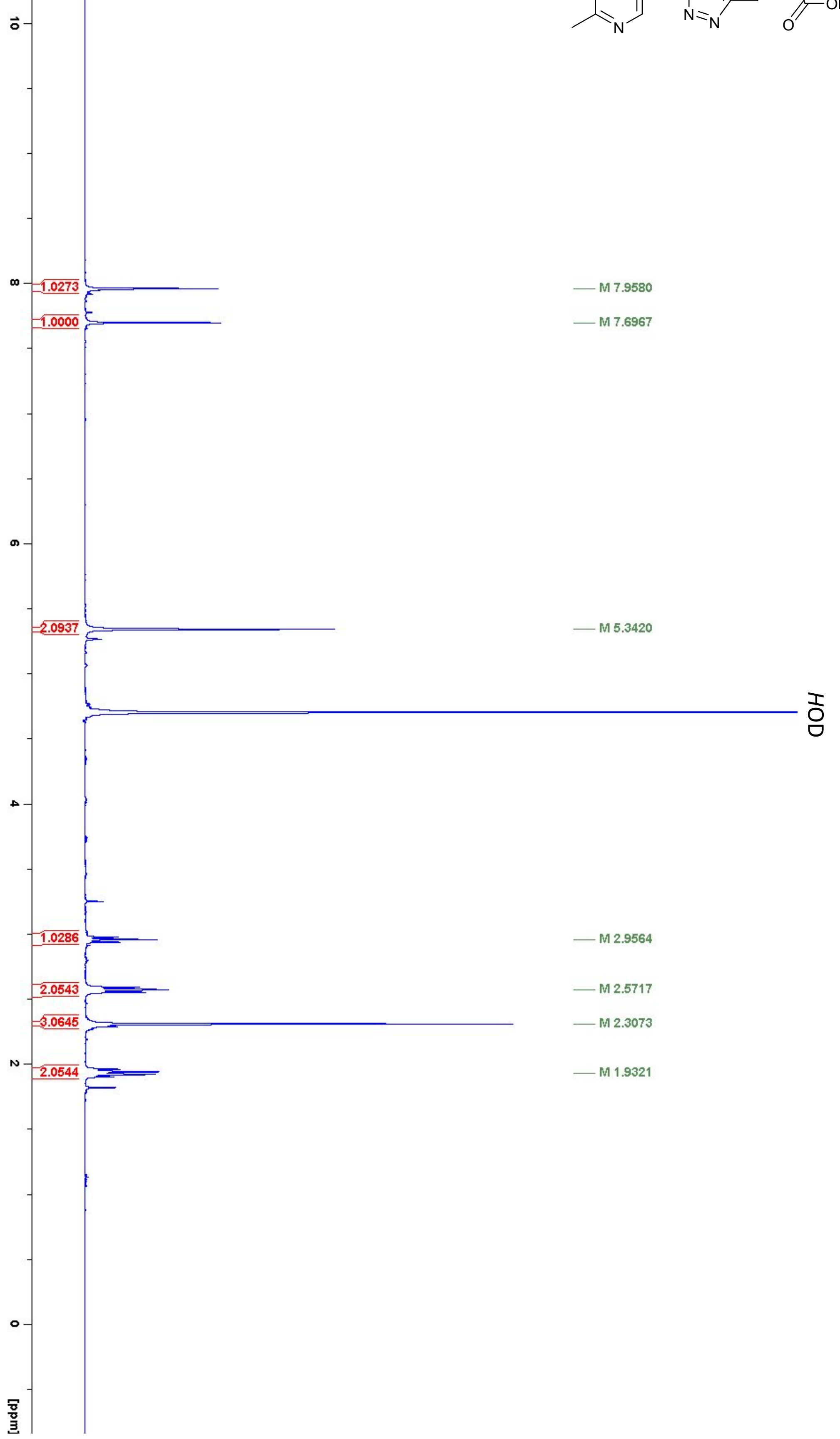

^{13}C NMR of **14a** in D_2O:

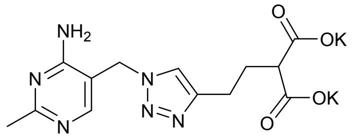

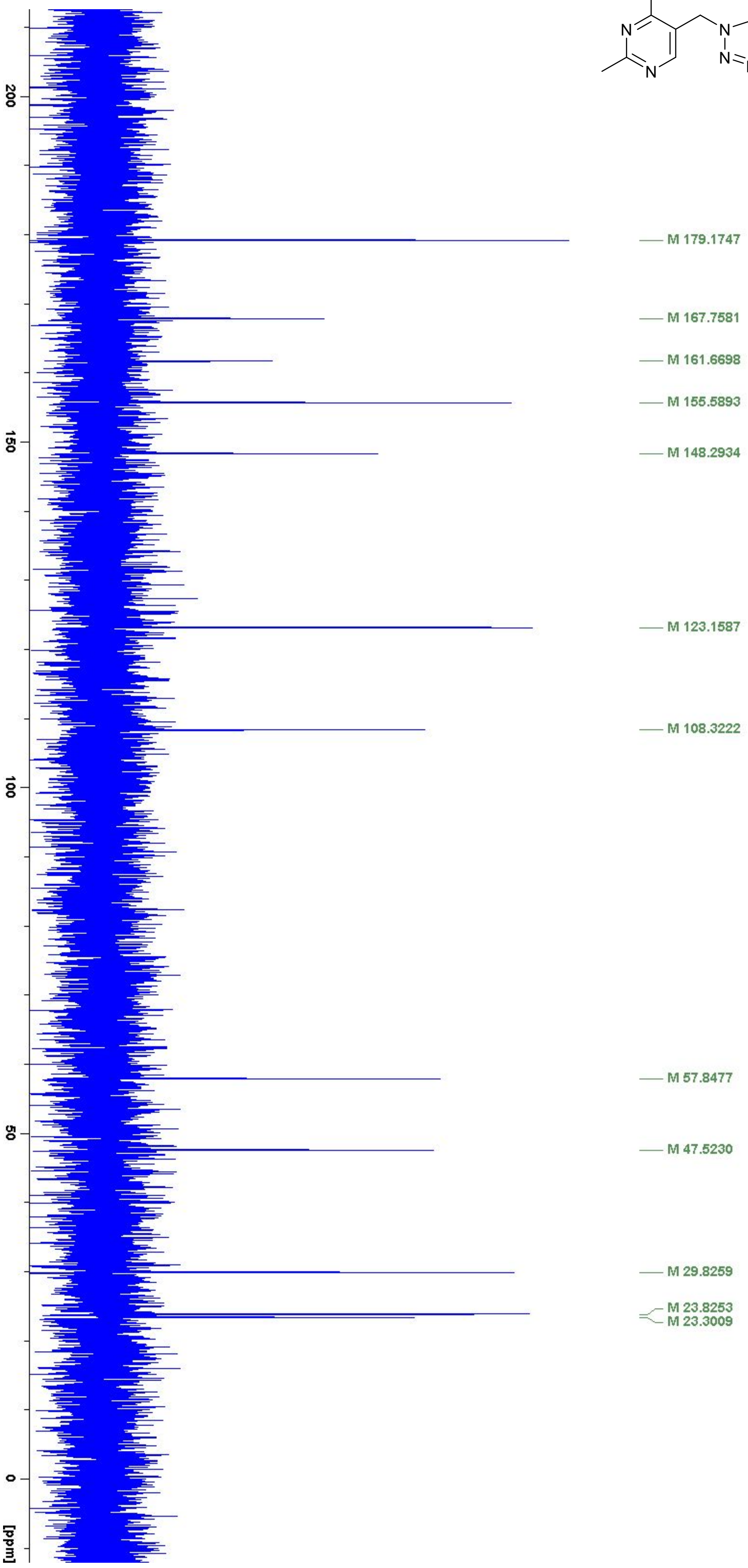

^{1}H NMR of **14b** in D_2O:

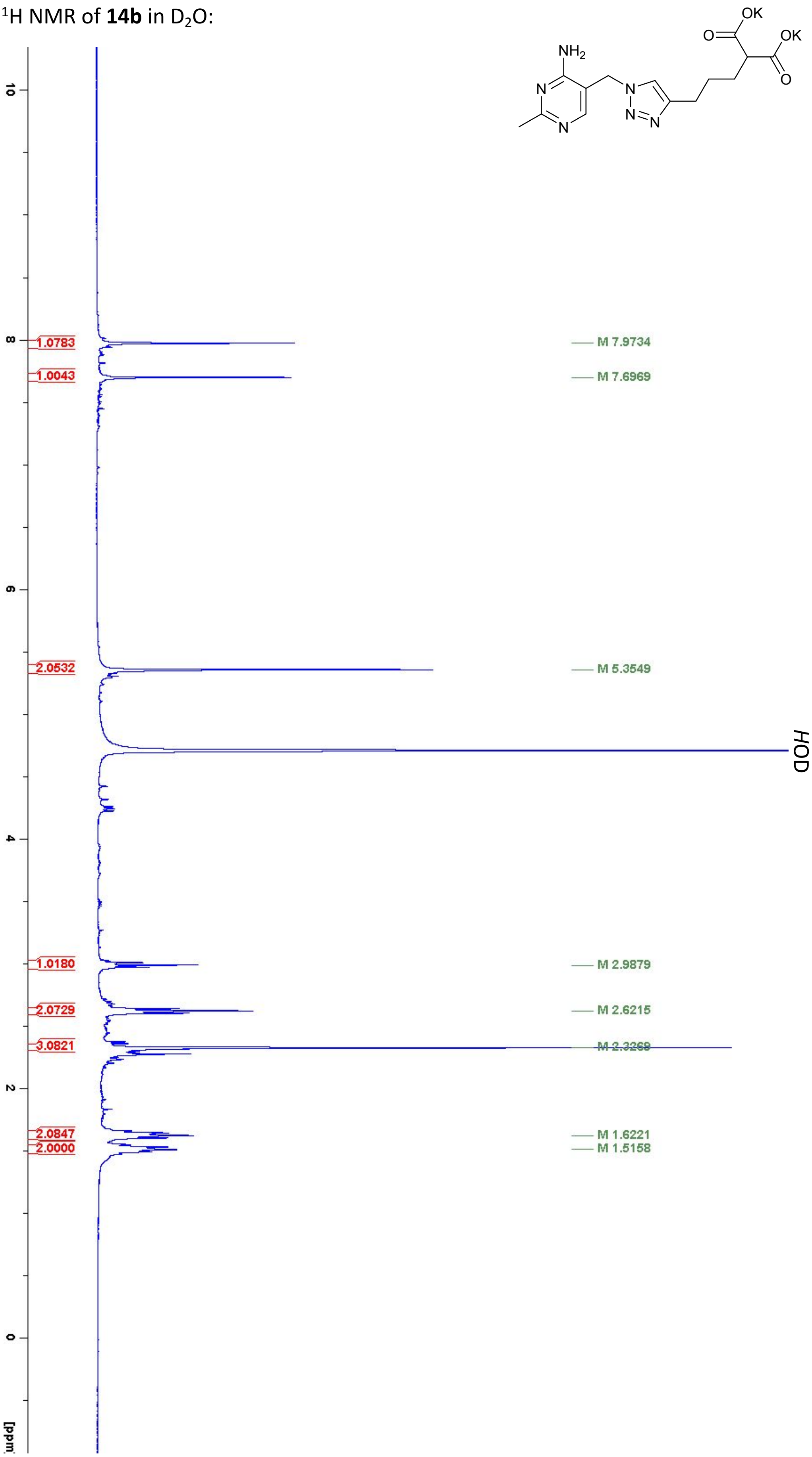

^{13}C NMR of **14b** in D_2O:

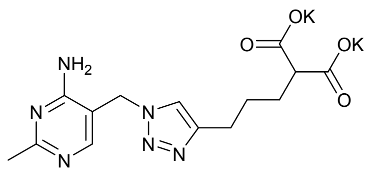

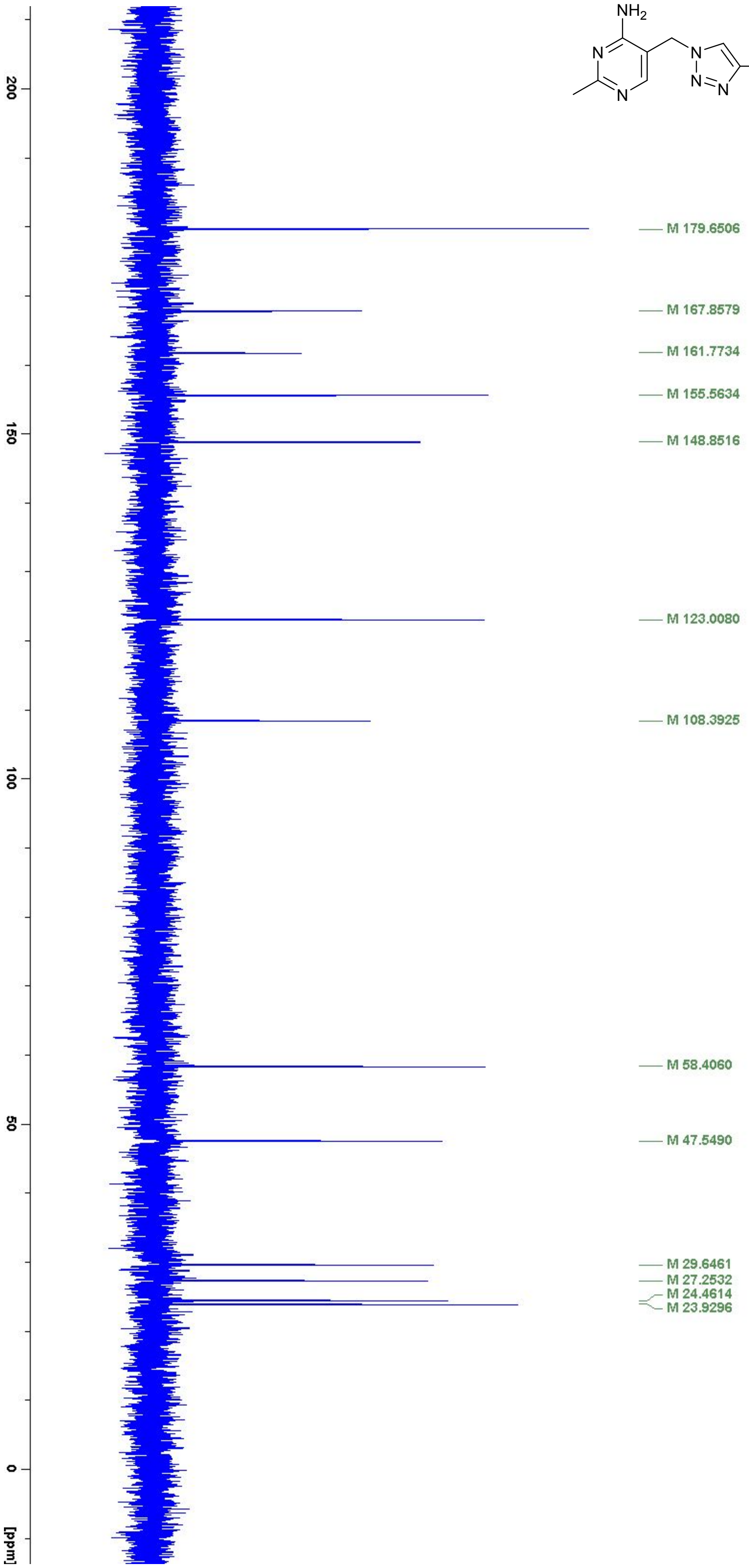

^{1}H NMR of **15a** in CD_3OD:

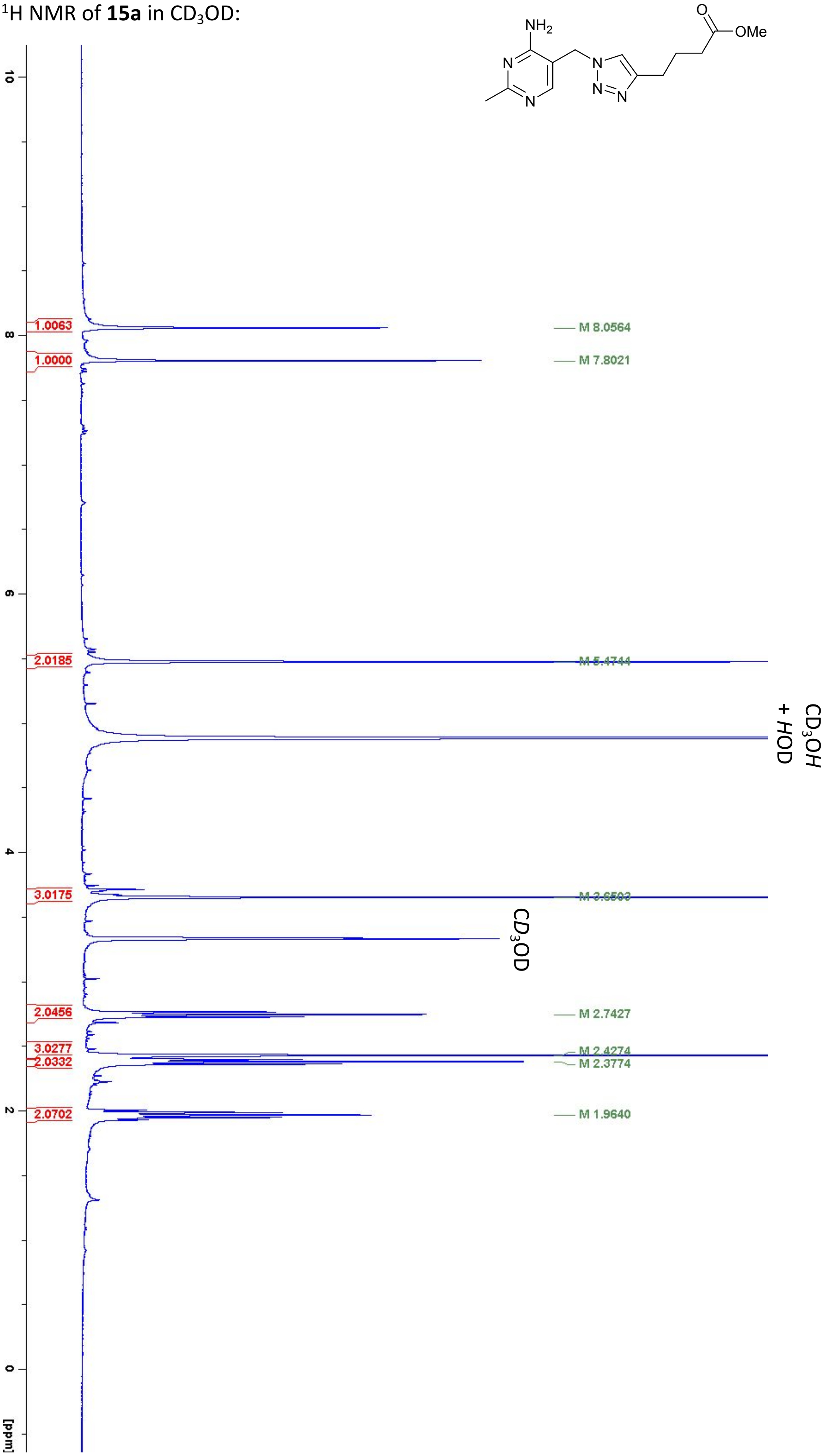

^{13}C NMR of **15a** in CD_3OD:

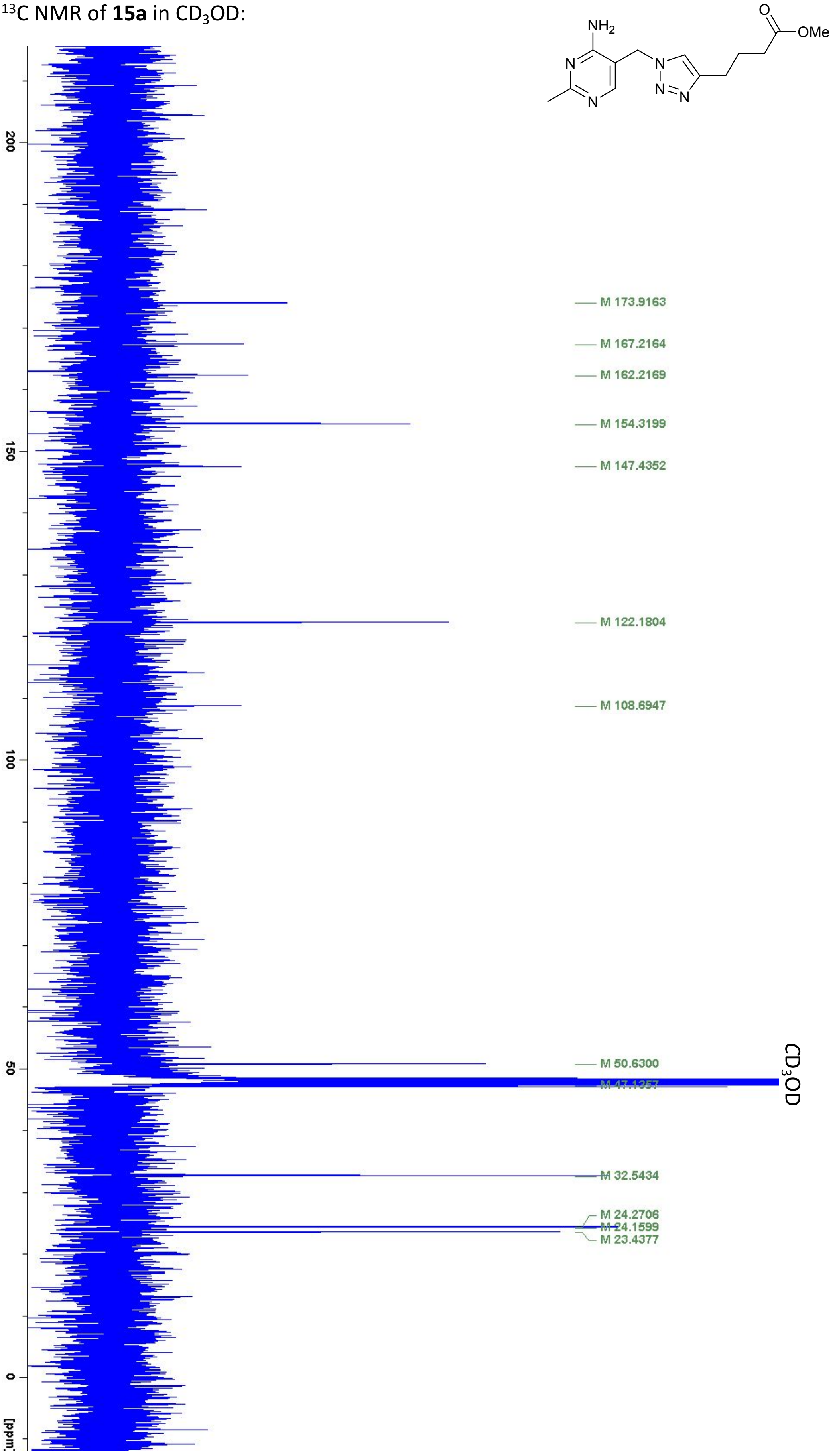

^{1}H NMR of **15b** in CD_3OD:

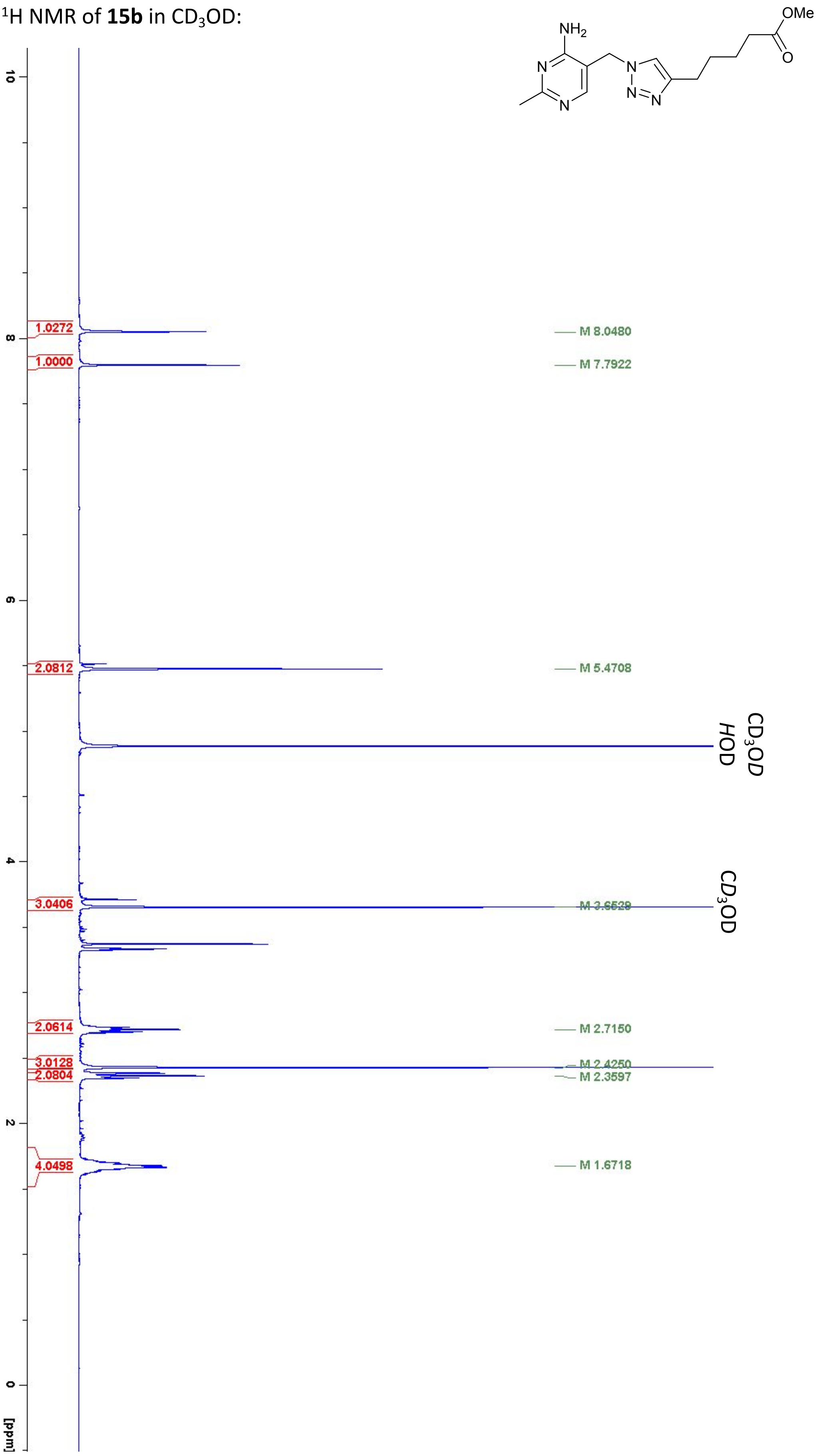

^{13}C NMR of **15b** in CD_3OD:

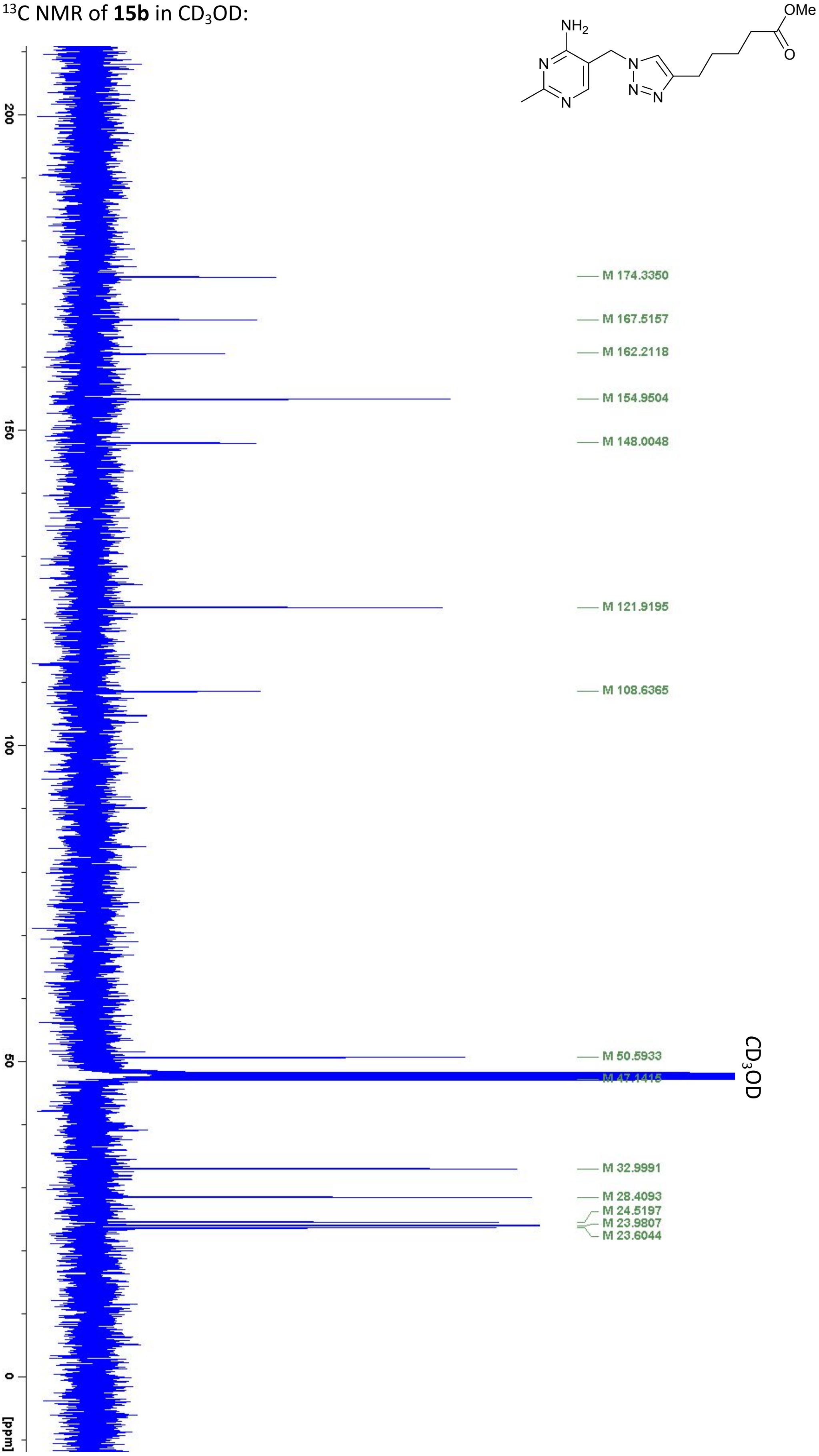

^{1}H NMR of **16a** in CD_3OD:

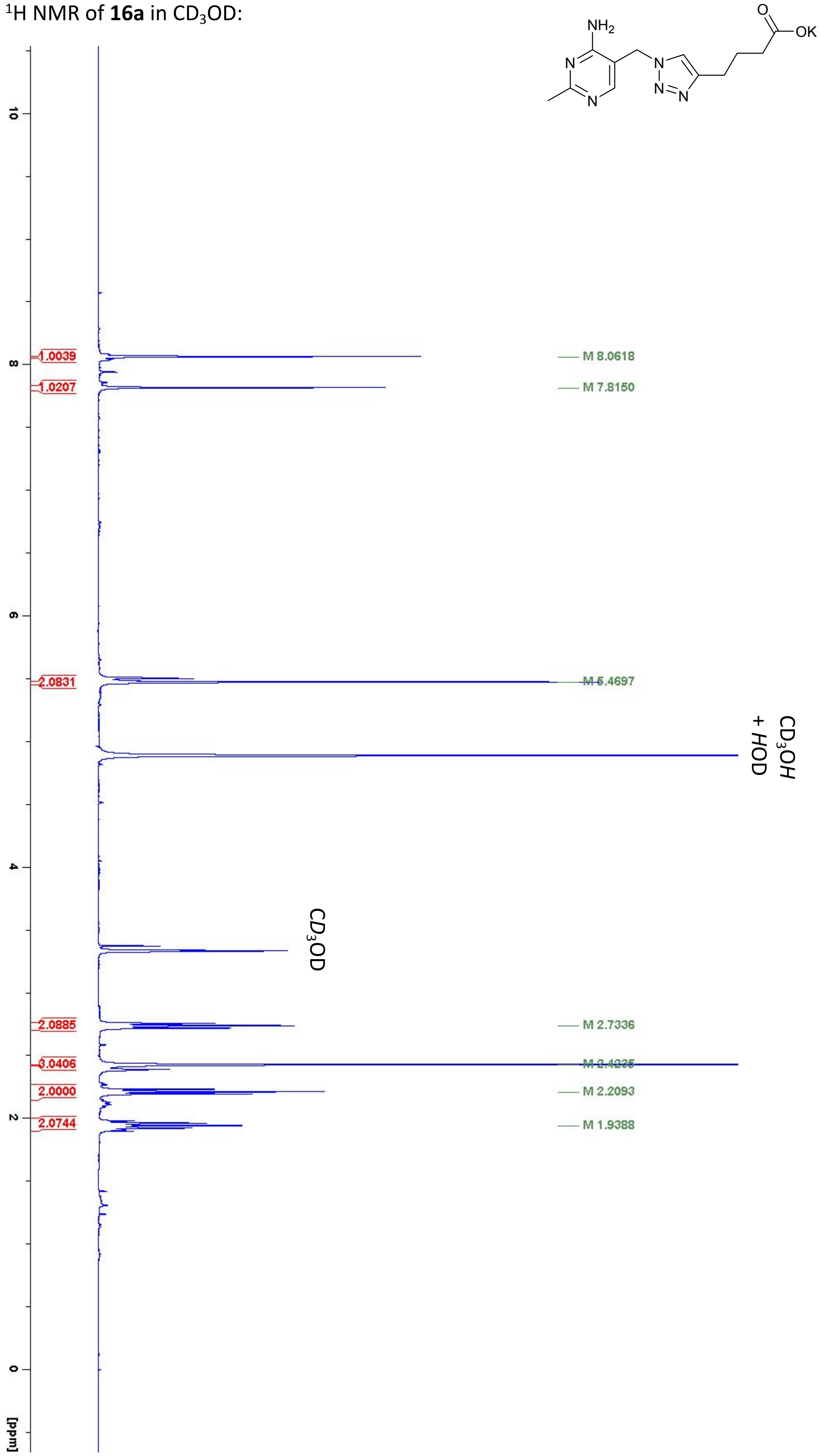

^{13}C NMR of **16a** in CD_3OD:

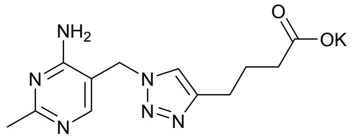

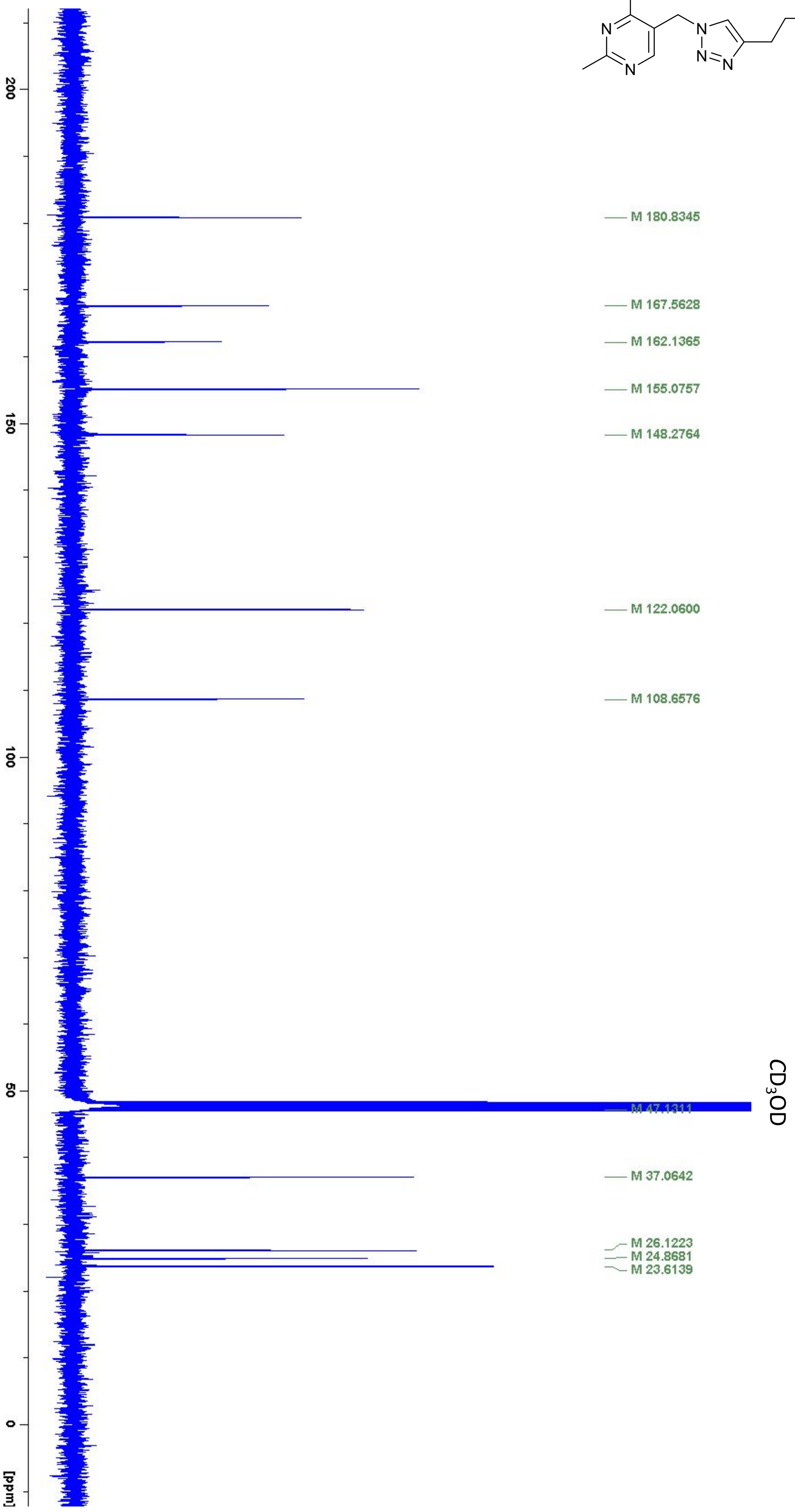

^{1}H NMR of **16b** in D_2O:

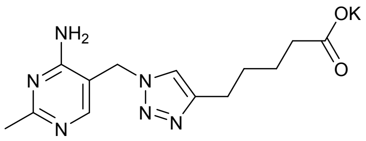

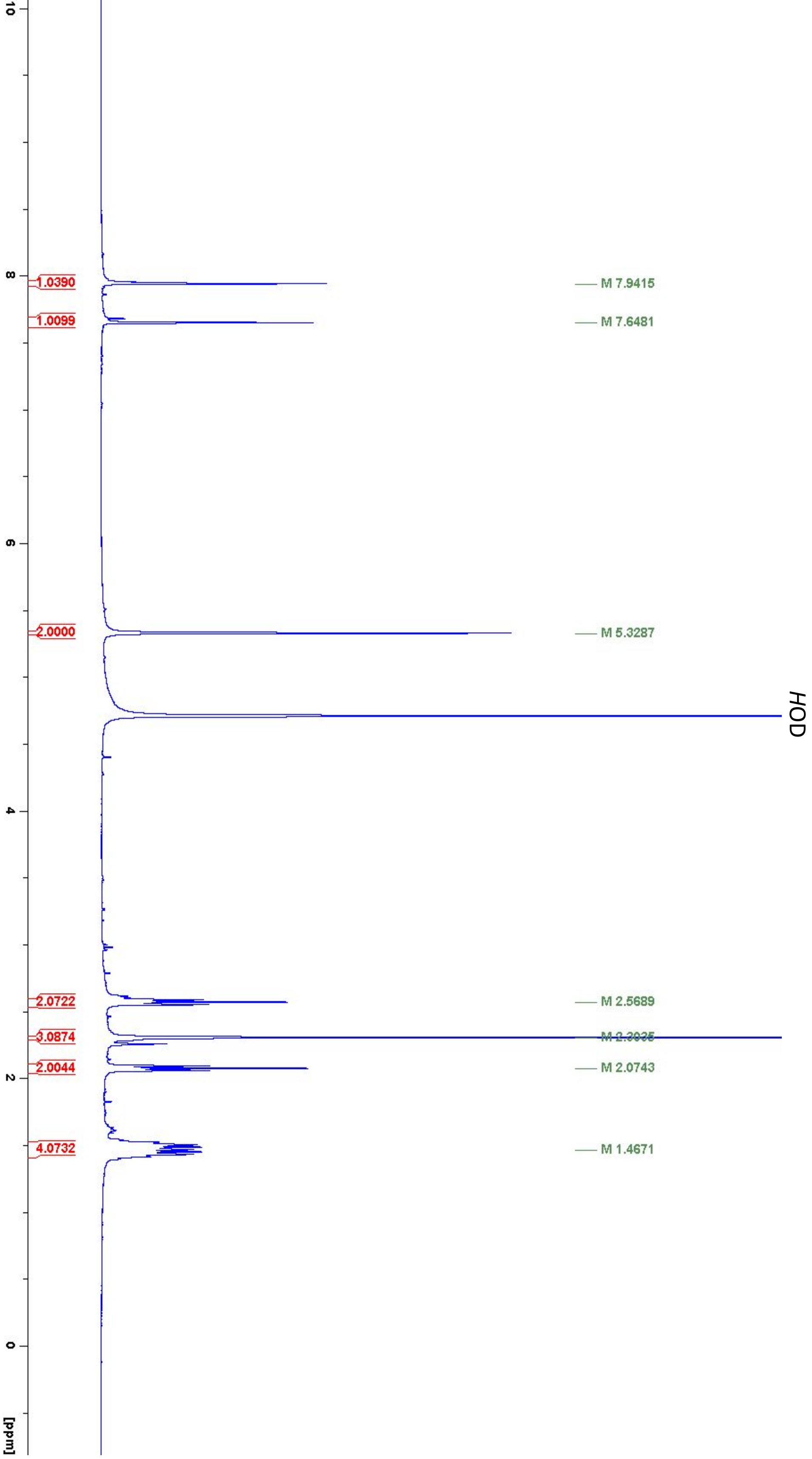

^{13}C NMR of **16b** in D_2O:

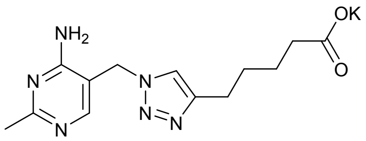

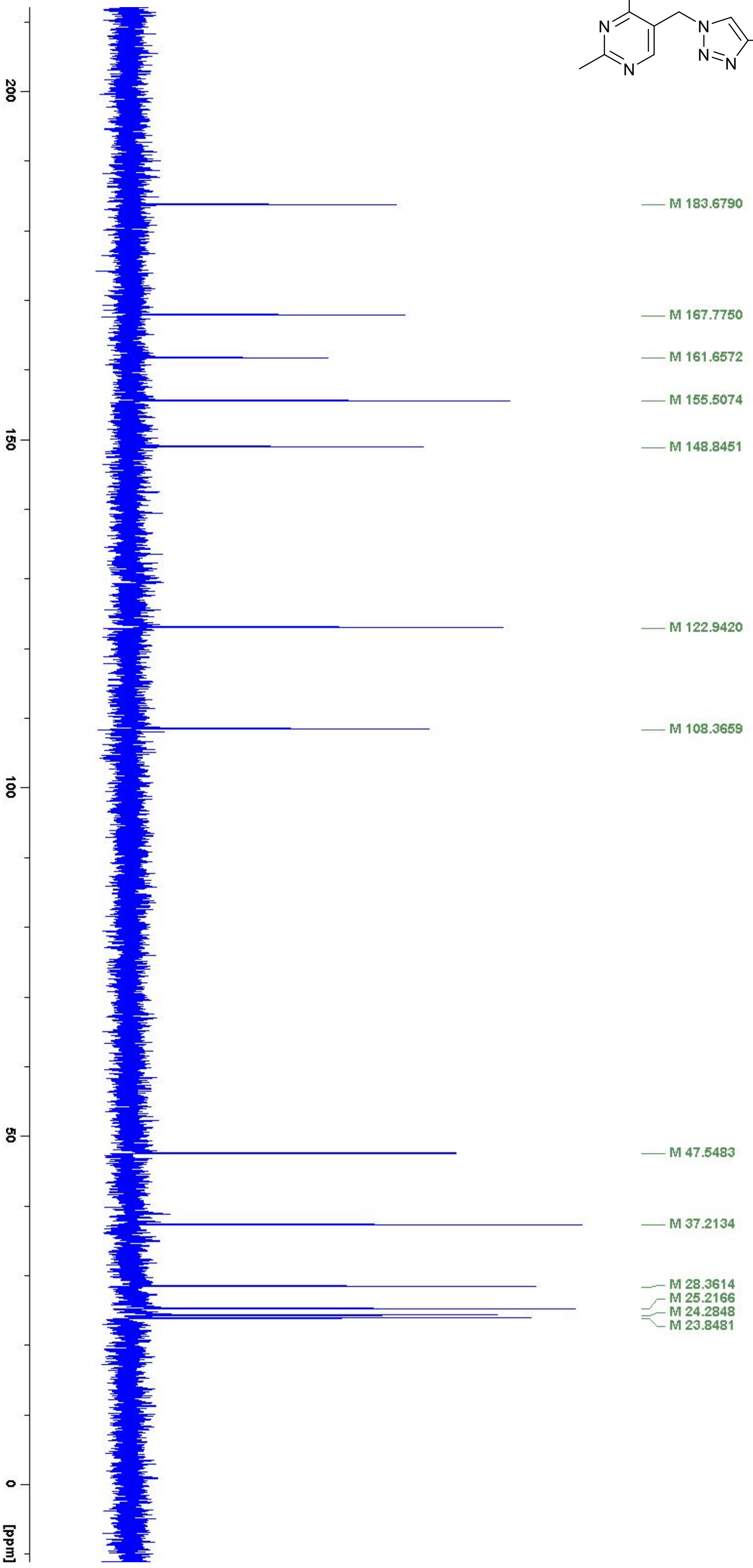

References

1. Wohnsland, F.; Faller, B. High-Throughput Permeability pH Profile and High-Throughput Alkane/Water Log *P* with Artificial Membranes. *J. Med. Chem.* **2001**, *44* (6), 923–930. https://doi.org/10.1021/jm001020e.
2. Zhu, C.; Jiang, L.; Chen, T.-M.; Hwang, K.-K. A Comparative Study of Artificial Membrane Permeability Assay for High Throughput Profiling of Drug Absorption Potential. *Eur. J. Med. Chem.* **2002**, *37* (5), 399–407. https://doi.org/10.1016/S0223-5234(02)01360-0.
3. Chan, A. H. Y.; Ho, T. C. S.; Irfan, R.; Hamid, R. A. A.; Rudge, E. S.; Iqbal, A.; Turner, A.; Hirsch, A. K. H.; Leeper, F. J. Design of Thiamine Analogues for Inhibition of Thiamine Diphosphate (ThDP)-Dependent Enzymes: Systematic Investigation through Scaffold-Hopping and C2-Functionalisation. *Bioorg. Chem.* **2023**, *138*, 106602. https://doi.org/10.1016/j.bioorg.2023.106602.
4. Jahn, B.; Jonasson, N. S. W.; Hu, H.; Singer, H.; Pol, A.; Good, N. M.; den Camp, H. J. M. O.; Martinez-Gomez, N. C.; Daumann, L. J. Understanding the Chemistry of the Artificial Electron Acceptors PES, PMS, DCPIP and Wurster's Blue in Methanol Dehydrogenase Assays. *J. Biol. Inorg. Chem.* **2020**, *25* (2), 199–212. https://doi.org/10.1007/s00775-020-01752-9.
5. Merk, D.; Grisoni, F.; Friedrich, L.; Gelzinyte, E.; Schneider, G. Computer-Assisted Discovery of Retinoid X Receptor Modulating Natural Products and Isofunctional Mimetics. *J. Med. Chem.* **2018**, *61* (12), 5442–5447. https://doi.org/10.1021/acs.jmedchem.8b00494.
6. Perez, C.; Daniel, K. B.; Cohen, S. M. Evaluating Prodrug Strategies for Esterase-Triggered Release of Alcohols. *ChemMedChem* **2013**, *8* (10), 1662-1667. https://doi.org/10.1002/cmdc.201300255.
7. Chan, A. H. Y.; Ho, T. C. S.; Fathoni, I.; Pope, R.; Saliba, K. J.; Leeper, F. J. Inhibition of Thiamine Diphosphate-Dependent Enzymes by Triazole-Based Thiamine Analogues. *ACS Med. Chem. Lett.* **2023**, *14* (5), 621–628. https://doi.org/10.1021/acsmedchemlett.3c00047.
8. Siegel, D.; Hui, H. C.; Doerffler, E.; Clarke, M. O.; Chun, K.; Zhang, L.; Neville, S.; Carra, E.; Lew, W.; Ross, B.; Wang, Q.; Wolfe, L.; Jordan, R.; Soloveva, V.; Knox, J.; Perry, J.; Perron, M.; Stray, K. M.; Barauskas, O.; Feng, J. Y.; Xu, Y.; Lee, G.; Rheingold, A. L.; Ray, A. S.; Bannister, R.; Strickley, R.; Swaminathan, S.; Lee, W. A.; Bavari, S.; Cihlar, T.; Lo, M. K.; Warren, T. K.; Mackman, R. L. Discovery and Synthesis of a Phosphoramidate Prodrug of a Pyrrolo[2,1-*f*][Triazin-4-Amino] Adenine *C* -Nucleoside (GS-5734) for the Treatment of Ebola and Emerging Viruses. *J. Med. Chem.* **2017**, *60* (5), 1648–1661. https://doi.org/10.1021/acs.jmedchem.6b01594.
